# New Palaearctic species of the tribe Thalassaphorurini Pomorski, 1998 (Collembola, Onychiuridae)

**DOI:** 10.3897/zookeys.126.1229

**Published:** 2011-09-02

**Authors:** Anatoly B. Babenko, Ayuna B. Chimitova, Sophya K. Stebaeva

**Affiliations:** 1 Severtsov Institute of Ecology & Evolution, Russian Academy of Sciences, Leninski pr. 33, Moscow 119071; 2 Moscow State Pedagogical University, Kibalchich str. 6, korp. 5, Moscow 129164; 3 Institute of Systematics & Ecology of Animals, Siberian Branch of Russian Academy of Sciences, Frunze str. 11, Novosibirsk 630091

**Keywords:** ά-taxonomy, morphological review, *Sensillonychiurus*, *Allonychiurus*, northern Asia, eastern Europe

## Abstract

The paper is devoted to a taxonomic revision of the genus *Sensillonychiurus* Pomorski & Sveenkova, 2006. Five new species of this genus, i.e. *Sensillonychiurus mirus* **sp. n.**, *Sensillonychiurus taimyrensis* **sp. n.**, *Sensillonychiurus vegae* **sp. n.**, *Sensillonychiurus vitimicus* **sp. n.**, and *Sensillonychiurus amuricus* **sp. n.**, as well as three new species of the related genus *Allonychiurus* Yoshii, 1995, i.e. *Allonychiurus subvolinensis* **sp. n.**, *Allonychiurus elikonius* **sp. n.**, and *Allonychiurus unisetosus* **sp. n.** are being described from various regions of Eurasia. The diagnoses of both genera are amended to include described species. Two genera, *Tantulonychiurus* Pomorski, 1996 and *Thibaudichiurus* Weiner, 1996, are treated as junior synonyms of the genus *Allonychiurus*. *Agraphorura eisi* (Rusek, 1976) is transferred to *Sensillonychiurus*; *Tantulonychiurus volinensis* (Szeptycki, 1964) and *Tantulonychiurus asiaticus* Babenko, 2007 to *Allonychiurus*. A review of morphological peculiarities of *Sensillonychiurus* is performed, comparisons with the other genera of Thalassaphorurini given, and a key to the known species provided.

## Introduction

This paper has been prompted through the discovery of a new species on the Barents coast of Kola Peninsula. This species from the tribe Thalassaphorurini is characterized by the combination of morphological features that fails to completely fit into any of the known genera of the tribe. Unfortunately, the tribe’s generic classification, as well as that of the whole subfamily Onychiurinae, is still far from perfect. Starting from the pioneering papers by Bagnall (1948, 1949) and until recently, about 15 genera have been proposed for various representatives of the tribe. Most of these genera are entirely valid, yet some are considered junior synonyms. For instance, at least four synonyms are known for the type genus of the tribe, *Thalassaphorura* Bagnall, 1949 alone (see Sun et al. 2010). At present, according to the database of Collembola of the World (Bellinger et al. 1996–2011) the tribe includes nine widely accepted genera: *Thalassaphorura*, *Micronychiurus* Bagnall, 1949, *Uralaphorura* Martynova, 1978, *Allonychiurus* Yoshii, 1995, *Spinonychiurus* Weiner, 1996, *Tantulonychiurus* Pomorski, 1996, *Agraphorura* Pomorski, 1998, *Detriturus* Pomorski, 1998, and *Sensillonychiurus* Pomorski & Sveenkova, 2006. In addition, there are several generic names that are only used occasionally. Thus, the provisional synonymy of *Thibaudichiurus* Weiner, 1996 with *Allonychiurus* was recently rejected by Sun et al. (2011).

The only character uniting all of the members of the tribe Thalassaphorurini is the structure of the furcal remnant which forms a finely granulated area in mid-section of *Abd*.4 with 4 small setae arranged in two posterior rows. The second character shared, i.e. distinct antennal and tergal sensilla, is probably present in all genera, but not all species of the tribe. Taking this into account, evidently the genus *Uralaphorura* is to be excluded from the tribe in having nothing in common with the other Thalassaphorurini, being characterized by a quite different structure of the furcal remnant with four posterior setae arranged in a line. Thus, *Uralaphorura* is probably closer to Onychiurini than to Thalassaphorurini (see also Babenko 2009).

According to R.J. Pomorski (personal communication), two of the remaining eight genera, i.e. *Micronychiurus* and *Agraphorura* are to be considered as synonyms. Nevertheless we do not follow here this suggestion as it was never officially published and a discussion on the status of these genera is beyond the scope of our paper. In his draft Synopsis on Palaearctic Onychiuridae, Pomorski also intended to synonymize the genus *Tantulonychiurus* (and also *Thibaudichiurus*) with *Allonychiurus*. Most probably, this latter suggestion was dictated by its practical usefulness, as well as by the impossibility to unity the known species of all these “genera” into more or less natural groups, based only on our present knowledge. For instance, according to Sun et al. (2009) only five of about two dozen known species of *Allonychiurus* completely correspond to the diagnoses as given by Yoshii (1995) and Weiner (1996). Later the generic diagnosis was amended by Sun et al. (2011) to include some species showing a partly reduced tibiotarsal chaetotaxy and smooth sensillar clubs in *AO*. In this scope, *Allonychiurus* differs from both *Thibaudichiurus* and *Tantulonychiurus* only in having several rows of manubrial setae posterior to a furcal remnant, and more numerous labral setae. Two latter genera were separated by the position of *MVO* and a different number of distal setae on the tibiotarsi, namely seven setae in two so far known species of *Tantulonychiurus* whereas the type species of the genus *Thibaudichiurus* has not eleven (Sun et al. 2011) but nine setae in distal whorl (personal communication of L. Deharveng). Such a generic classification of the complex partly simplified the situation, but did not completely solve the main problem existing in the group, i.e., the existence of many inadequately described species which can only provisionally be placed in this or that modern genus. Thus, six of 14 species treated as representatives of the genus *Allonychiurus* in the last paper of Sun et al. (2011) were marked by an asterisk indicating that “*species assignment requires confirmation*”. Furthermore, it is rather difficult to apply this division to some known species as well, since some are purely parthenogenetic or just lack modified setae in reproductive males, e.g. *Tantulonychiurus asiaticus* Babenko, 2007, which is in other respects virtually identical to the members of the *Thibaudichiurus*/*Tantulonychiurus* complex. The degree of reduction of the tibiotarsal setae appears to be a rather variable character in some genera of Thalassaphorurini, still being unknown for many described forms. As a revision of all these related genera is beyond the scope of the present paper, we tentatively accept here a broadened conception of *Allonychiurus* (see diagnosis in the end of the paper) and use the following generic classification of Thalassaphorurini as consisting of seven genera: *Spinonychiurus* (*d*0 on head absent, *PAO* lobes compound, sternum of *Abd*.3 clearly subdivided, tibiotarsi with 11 setae in distal whorl), *Detriturus* (*d*0 absent, *PAO* lobes compound, sternum of *Abd*.3 not subdivided, *Abd*.5–6 fused, tibiotarsi with 11 setae in distal whorl), *Sensillonychiurus* (*d*0 absent, *PAO* lobes compound, sternum of *Abd*.3 not subdivided, *Abd.*5–6 clearly separated, tibiotarsi with 7 or 9 distal setae), *Thalassaphorura* (*d*0 present, *PAO* lobes simple), *Micronychiurus* (*d*0 present, *PAO* lobes compound, *Abd*.6 with 1+1 prespinal microsetae, multiplication and unusual position of anterior *pso* on head and on abdominal tip, *AS* present or absent), *Agraphorura* (*d*0 present, *PAO* lobes compound, *Abd*.6 with 1+1 prespinal microsetae, low number of dorsal *pso* in usual position, *AS* absent) and *Allonychiurus* (*d*0 present, *PAO* lobes compound, *Abd*.6 with 2+2 prespinal setae, *AS* present). The latter genus includes two rather distinct species-groups, the *flavescens*-group and the *volinensis*-group, which clearly differ habitually, as well as in the number of labral setae and setal rows in the manubrial zone of *Abd*.4, but both latter characters may be size-dependent. At the same time, we are sure that this generic system requires additional attention, while the scopes of some genera might be cardinally changed in future.

The new species mentioned in the beginning of Introduction appears to be especially similar to the known representatives of the small eastern Asiatic genus *Sensillonychiurus*. A study of the available material from M. Potapov’s and authors’ collections reveals a whole number of closely related forms and shows that the original diagnosis of the genus must be somewhat amended. Thus, the present paper includes a brief review of the morphological peculiarities of *Sensillonychiurus* as compared to the other genera of the tribe, a slightly changed diagnosis and a key to all of the known species of this genus, as well as descriptions of five new species. In addition, three further new species habitually similar but, according to the accepted system of Thalassaphorurini, assignable to the genus *Allonychiurus*,have also been described and used for comparative purposes. Types of all the new species are deposited in the collection of the Department of Zoology & Ecology, Moscow State Pedagogical University (MSPU).

**Abbreviations**

A–E **papilla,** a, b, d, e **guards** – main labial papillae and associated guard setae (Fjellberg 1999)

A, AB, AC and ABC – four types of labium in Onychiuridae in accordance with the presence of thickened and blunt-tipped setae on corresponding labial papillae (Fjellberg 1999)

Abd**.1–6** – abdominal segments

A-B**,** T-setae, setae M and Y – tibiotarsal setae (Deharveng 1983)

Ant**.1–4** – antennal subsegments

AO – antennal organ on Ant.3

AS – anal spines on Abd.6

bl. f. – basolateral field of labium (mentum)

bm. f. – basomedial field of labium (submentum)

d**0** – unpaired axial seta on area frontalis of the head

a**0**, m**0** and p**0** – unpaired axial setae on terga

Lg.**1–3** – legs

ms – microsensillum

MSPU – Moscow State Pedagogical University

MVO – male ventral organ

PAO – postantennal organ

pso – pseudocellus

psx – parapseudocellus

px – proximal setae on labium

Th**.1–3** – tergal segments

Ti**.1–3** – tibiotarsi

U**3** – inner edge of unguis on hind leg

VT – ventral tube

## A review of the main morphological characters of Sensillonychiurus Pomorski & Sveenkova, 2006

The present review is based on the morphological peculiarities of five new species described in this paper, as well as on published data on all four so far known species of the genus. Three of them were described by the authors of the genus (Pomorski and Sveenkova 2006), the fourth one, *Sensillonychiurus eisi* (Rusek, 1976), comb. n., has hitherto been treated as a species of *Agraphorura* (see Pomorski 2004; Arbea 2005). Nevertheless, there is good evidence of its similarity to *Sensillonychiurus*, for instance, in the absence of *d*0 on the head and in the presence of only three guard setae in *AO* (see figs 11A and 12D in Rusek 1976).

*Body shape and size.* All of the so far known species of *Sensillonychiurus* are among the smallest Onychiurinae, with body size ranging between 0.4 and 0.7 mm. The body is slender and elongated (Figs 8–9), with rather short antennae and clearly club-shaped *Ant*.4 (Fig. 10). Area antennalis is not distinctly demarcated.

**Figures 1–7.**
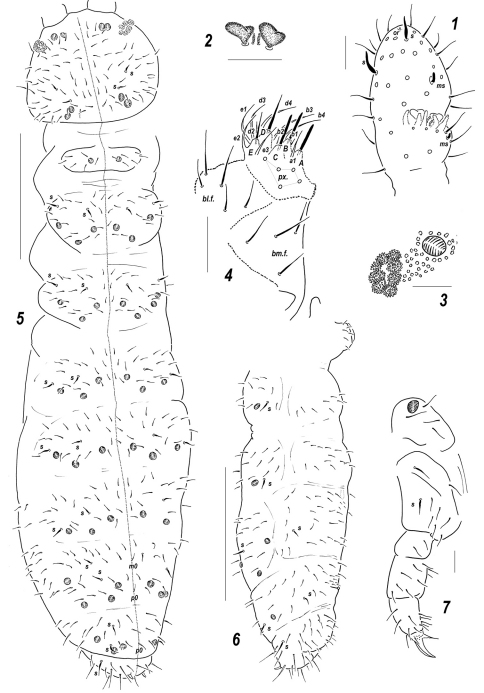
*Sensillonychiurus mirus* sp. n. **1** *Ant*.3–4; **2** sensorial elements of *Ant*.3 organ **3** *PAO* and adjacent *pso* **4** labium **5** dorsal chaetotaxy and *pso* distribution **6** abdomen, lateral view **7** *Lg*.3. Scales: 5–6 – 0.1 mm, 1–4, 7 – 0.01 mm.

*Sensillar armature of the antennae*. Pomorski and Sveenkova (2006) considered the presence of only three guard setae in *AO* as the main diagnostic feature of the genus *Sensillonychiurus*. These authors, based on an examination of all three species then known, found this character as being unique not only to Thalassaphorurini, but to all other Onychiurinae as well. They wrote that it “*may indicate that the new taxon is monophyletic*”. However, our study reveals that not all of those species, albeit indeed strikingly similar, are characterized by such a deep reduction of the number of guard setae in *AO*. Thus, a far more usual number (4) of guard setae was found in the European *Sensillonychiurus mirus* sp. n., as well as in two eastern Asian species, *Sensillonychiurus vitimicus* sp. n.and *Sensillonychiurus amuricus* sp. n., thus correlating with a full set (5) of papillae. Only one congener, *Sensillonychiurus geminus* Pomorski & Sveenkova, 2006, has *AO* with five papillae but three guard setae. That is why not only the diagnosis of the genus has to be slightly amended, but its distinctions from the other genera of the tribe must be reconfirmed, although most of Thalassaphorurini are characterized by a complete set (5) of guard setae in *AO*. Apart from *Sensillonychiurus*, species with less numerous (4) guard setae are known only among *Agraphorura*. Discarding this character, the sensillar chaetotaxy of the antennae in *Sensillonychiurus* is not genus-specific, being more or less similar to that in the other genera of Thalassaphorurini: *Ant*.4 always bears two distinct thickened sensilla (a dorsal one subapically and an inner one in the mid-section of the segment), a small subapical organite (*or*) and a subbasal microsensillum (*ms*) which is clearly larger than that on *Ant*.3 (see, for instance, Figs 1, 10). The latter character is also typical of *Micronychiurus* and *Agraphorura*. The position of *ms* on *Ant*.4 in relation to ordinary setae slightly varies between different species (cf. Figs 11–14 and 15–17) and can be used in their identification. Sensorial elements in *AO* of different species of the genus are similar: clubs are smooth, more or less roundish, with or without clear ribs. A different type of sensorial clubs in *AO* (distinctly granulated, morula-like) is known among Thalassaphorurini only in some *Thalassaphorura* and in the *flavescens*-group of *Allonychiurus*.

*Structure of the PAO*. All species of *Sensillonychiurus* show a relatively wide *PAO* consisting of few (6–8) vesicles with numerous secondary lobes. As a whole, it usually looks like a single mass with only traces of vesicle divisions (Fig. 3).

*Labrum*. All congeners are characterized by a constant number (7) of labral setae, four distal ones being longer and clearly thicker, and two or four prelabral setae. The variant with two prelabral setae seems to be more common (see Table 1), but this character is still unknown in *Sensillonychiurus eisi*, *Sensillonychiurus virginis* Pomorski & Sveenkova, 2006 and *Sensillonychiurus geminus*. Such a slightly reduced number of labral setae is also typical of all *Thalassaphorura* known for this character, as well as of the *volinensis*-group of *Allonychiurus*, but not of the *flavescens*-group, at least some of which showing nine labral setae (Yoshii 1995; Sun et al. 2009, 2011). This feature is completely unknown in *Detriturus*, *Spinonychiurus*, and *Micronychiurus*, whereas among *Agraphorura* the existing information concerns only *Agraphorura calvoi* Arbea, 2005, which has nine labral setae (a presumed basal set for Onychiurinae), and *Agraphorura sangelensis* Kaprus’ & Stebaeva, 2006, with two prelabral and seven labral setae (our data).

*Labium*. The type of labium most frequently seen in the genus is *AC*, with the *ABC*-type is found only in two species, *Sensillonychiurus mirus* sp. n. and *Sensillonychiurus vitimicus* sp. n. The number of setae on the proximal, basal and laterobasal fields of the labium is more or less stable, although individual variations and some asymmetry are visible in some specimens. The number of distal guard setae of the labial palp corresponds to the most common (and also complete) set found in Onychiurinae (Fjellberg 1999): seven long guard setae (*b*3-4, *d*3-4, and *e1*-3) and four shorter (*a*1, *b*1-2 and *d*2) ones set on papillae. The only notable peculiarity of the labium in the study group is the unusual length of *a*1 seta which is clearly longer and thicker than *b*1-2 or *d*2 (Fig. 4). Unfortunately, the fine structure of the labium is known only for a few representatives of the tribe, this not allowing for serious comparisons to be made. We can only state that all three types of labium (*А*, *АС* and *АВС*) are known in *Thalassaphorura* (Sun et al. 2010), with *АС* being the most common. In the genus *Allonychiurus*, two types (*AC* and *ABC*) are found among species of the *volinensis-*group (Fjellberg 1999, our data), while only the *AC*-type is known in two species of the *flavescens-*group (see Sun et al. 2009, 2011). The *A*-type is observed in *Spinonychiurus epaphius* Kaprus’ & Tsalan, 2009 and, according to Pomorski and Sveenkova (2006), in the genus *Detriturus*. The *AB*-type seems to be most characteristic of the genera *Agraphorura* (Pomorski 2004; Arbea 2005; Kaprus’ and Stebaeva 2006) and *Micronychiurus* (Pomorski, pers. communication). The presence of a complete number of distal guard setae on the labial palp in such small-sized species as *Sensillonychiurus* is rather unexpected, as, for instance, all of the so far studied *Thalassaphorura* and members of the *volinensis*-group of *Allonychiurus*, being usually larger, have only ten guards (*e*2 absent) (Fig. 41). The same is probably characteristic of the *flavescens*-group of *Allonychiurus* (Sun et al. 2009, 2011) although the authors believe that not *e*2 but one of the *b*–setae is absent. A relatively long *a*1–seta could be suggested as a possible apomorphy of the genus, but there is not enough information concerning the other groups of Thalassaphorurini for such an assertion.

*Dorsal and ventral pso*. Contrary to the majority of Onychiurinae, the number of dorsal and ventral *pso* does not significantly vary within the genus, being almost always as following: 32/133/33343 (dorsal) and 1/000/0000 (ventral). There are only two exceptions: *Sensillonychiurus virginis*, with a lesser number of *pso* on thoracic terga(32/022/33343 as a whole), and *Sensillonychiurus geminus*, with some *pso* on two abdominal sterna. The ventral pseudocellar formula of the latter species was given differently by Pomorski and Sveenkova (2006) in the original description (1/000/0101) and in their comparative table of diagnostic characters (1/000/10010). The former version is probably the correct one. Apart from this, *Sensillonychiurusmirus* sp. n. often lacks the anteriormost *pso* of the postantennal group on a head. Such a dorsal formula (32/133/33343) is rather common in two other genera of Thalassaphorurini, namely, *Agraphorura* and *Allonychiurus*, known also in some *Thalassaphorura*, as well as in different genera of Onychiurini and Oligaphorurini. The absence of *pso* on abdominal sterna as the most usual character of *Sensillonychiurus* can also be found among *Spinonychiurus*, *Allonychiurus* and *Detriturus*.

*Parapseudocelli*. The complete absence of parapseudocelli (*psx*) on the subcoxae, femora and abdominal sterna is characteristic of most of the studied species of the genus, except for *Sensillonychiurus vegae* sp. n. which sometimes possesses a pair of *psx* on *Abd*.4. Such a weak development of *psx* is rather frequent among Thalassaphorurini, also known in *Micronychiurus*, *Agraphorura*, *Allonychiurus* (in both *flavescens*- and *volinensis*-groups),and some *Thalassaphorura*. Probably it at least partly correlates with the small size of specimens. Some intraspecific variations of *psx* numbers are likely (see, for instance, description of *Sensillonychiurus vegae* sp.n.) and need further attention.

**Figures 8–9.**
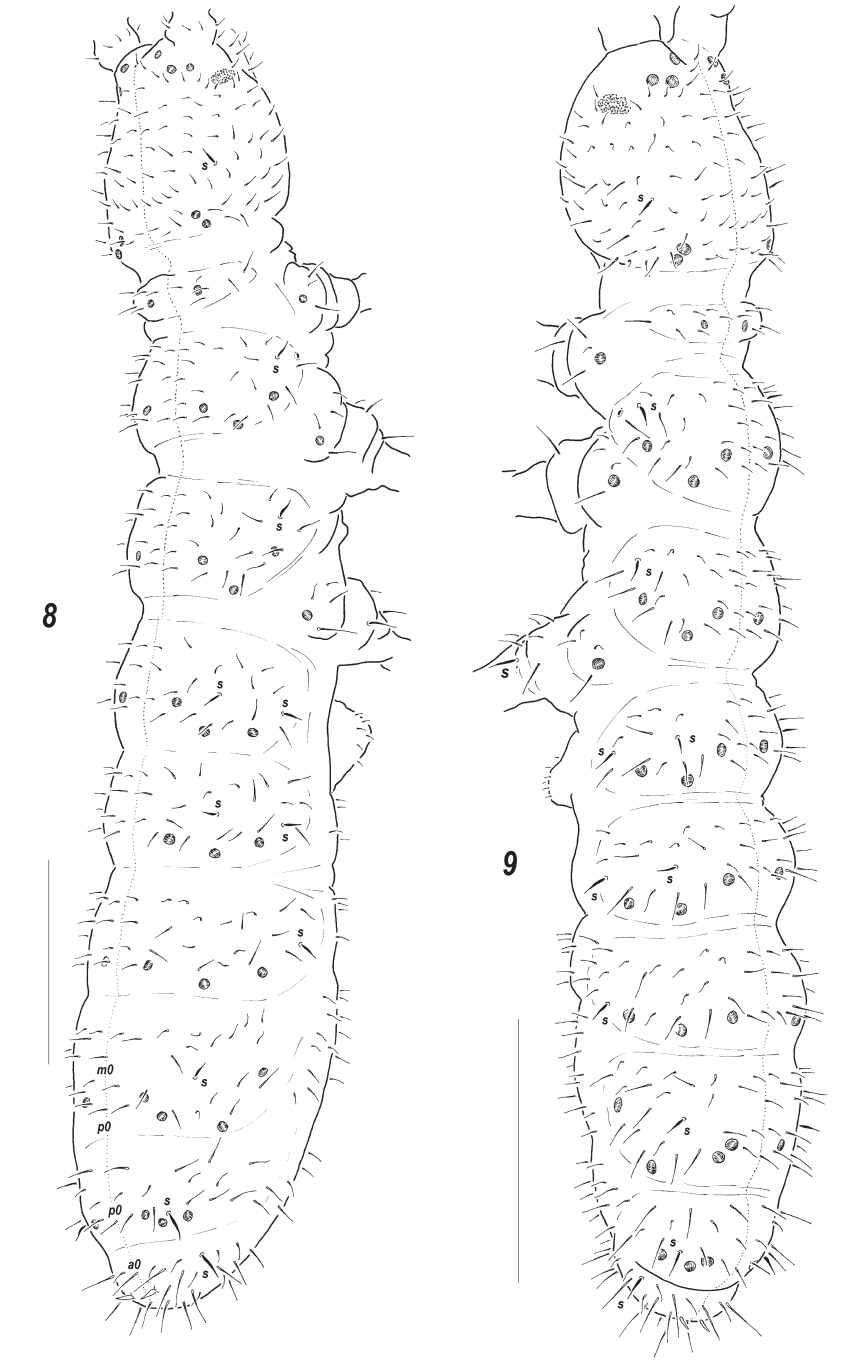
Dorsal chaetotaxy and *pso* distribution, *Sensillonychiurus taimyrensis* sp. n. **8** and *Sensillonychiurus vegae* sp. n. **9** Scale: 0.01 mm.

*Dorsal chaetotaxy.* The chaetotaxy in the genus was originally described as follows: “*Seta d0 on the head absent. Abdominal terga of IV, V and VI with 2, 1 and 1 medial setae, respectively*”. It can be added that these unpaired setae (*m*0 and *p*0 on *Abd*.4, *p*0 on *Abd*.5 and *a*0 on *Abd*.6) are meso- or macrosetae probably belonging to the primary chaetotic set, but not microsetae which can appear during ontogeny. Terga of *Th*.2-3 in adults with 3+3, of *Abd*.1-4 with 2+2 and of *Abd*.5 with 1+1, axial microsetae, additionally each tergum with 2+2 mesosetae in the axial group set out of line with microsetae (see, for instance, Fig. 8). The same pattern is found in all studied species which appear to have an almost symmetrical (especially in the mid-section of terga) and virtually identical dorsal chaetotaxy. This pattern seems to be unique to Thalassaphorurini. Thus, *Sensillonychiurus* shares the absence *d0* with only two genera of the tribe, *Spinonychiurus* and *Detriturus*. Known representatives of both these genera show different distributions of unpaired setae on the abdominal tip (Arbea and Jordana 1985; Palacios-Vargas and Diaz 1995; Pomorski 1998; Kaprus’ and Tsalan 2009), the most similar but yet not identical is that in *Detriturus jubilarius* (Gisin 1957) (see fig. 97G in Fjellberg 1998). In the group with *d*0 on the head, species of *Micronychiurus* and *Agraphorura* with known chaetotic patterns possess a medial seta only on *Abd*.6 (Palacios-Vargas and Deharveng 1982; Beruete et al. 1994; Pomorski 2004; Arbea 2005; Kaprus’ and Stebaeva 2006), *Allonychiurus* has quite a different chaetotaxy of *Abd*.6 with one or two medial setae and 2+2 prespinal microsetae (Figs 40, 49), unpaired setae on *Abd*.4 and 5 are microsetae if present (Lee 1973; Weiner 1989; Sun et al. 2009, 2011). A similar pattern is typical of most *Thalassaphorura*.

*Tergal and sternal sensilla.* The lateral microsensillum in all studied species is always present on *Th*.2, but usually absent from *Th*.3, except for two species, *Sensillonychiurus minusculus* Pomorski & Sveenkova, 2006 and *Sensillonychiurus geminus*. Several thickened macrosensilla in certain parts on terga and sterna are also very typical of Thalassaphorurini and of *Sensillonychiurus* as well. The most usual number of such sensilla in the studied species is as follows, 1/011/222111 from head to *Abd.*6 (Fig. 8), additionally two ventral sensilla are usually distinguishable on the anterolateral part of the head and one sensillum on each ventrolateral side of *Abd*.4 (Fig. 33). Variations are not frequent and somewhat obscure; the only clear exception being the European *Sensillonychiurus mirus* sp. n. which shows more dorsal sensilla (2/022/222221 as a whole). The described variability of the character in various genera of Thalassaphorurini permits to suggest that it can hardly be used in separating the genera. Moreover, the degree of sensillum differentiation varies widely both between and within species, being clearly age-dependent; sometimes the sensilla look like slightly thickened macrosetae distinguished only due to their positions. Some level of population variability of the character is not improbable either.

*Ventral chaetotaxy.* Most of the species of the genus lack setae on thoracic sterna. The only exception is *Sensillonychiurus vitimicus* sp. n., with 0-1-1 setae on each side of the *linea ventralis* on the thorax (Fig. 33). Among Thalassaphorurini, the complete absence of ventral setae on the thorax is only observed insome species of the genus *Micronychiurus* (Pomorski, pers. communication) and *Agraphorura* (Pomorski 2004; Arbea 2005). All studied species also show no setae at the base of *VT* and a rather stable number of setae on its distal lobes (usually 6+6). These latter characters are not unusual in Thalassaphorurini, known, e.g., in some *Micronychiurus*, *Agraphorura* and *Allonychiurus*.

*Tibiotarsal chaetotaxy* The pattern characteristic of all studied species of the genus can be described as follows: seven or nine setae in the distal whorl (all or two *T*-setae absent), 7-7-6 setae in *B*-whorl, *Y*-seta present, but *M*-seta absent (Figs 20, 29–30). The same pattern with 9 distal setae was previously found in *Sensillonychiurus eisi* by Fjellberg (1991). It is noteworthy that the latter character (absence of *M*-seta) only rarely occurs in Poduromorpha. Nevertheless, the same is probably characteristic of some *Agraphorura* (Palacios-Vargas and Deharveng 1982; Arbea 2005) but the number of tibiotarsal setae in the latter genus is rather variable, with both distal and proximal whorls being partly reduced. For instance, in *Agraphorura sangelensis* *Ti*.1-3 bare only 13-13-13 setae, respectively (seven in distal whorl, five *B*-setae and a slightly longer *Y*-seta set virtually in *B*-whorl, *M*-seta absent). Species of the genus *Micronychiurus* are known as having 7 or 9 distal setae and 8-8-7 setae in proximal whorls (Beruete et al. 1994), and so probably possess *M*-seta and lack one of the *B*-seta on *Ti*.3. All of the studied species of *Thalassaphorura*, as well as all *Allonychiurus* from the *volinensis*-group (also showing 7-9 distal setae on tibiotarsi), are characterized by a complete *B*-whorl on all legs (7-7-7) and the presence of both setae *M* and *Y* (Figs 47–48). The same pattern but with few additional proximal setae in *C*-whorls was known for *Allonychiurus antennalis* Sun, Chen & Deharveng, 2011 from the *flavescens*-group but the data for *Agraphorura megasomus* Sun, Yan & Chen, 2009 is different, with 11 distal setae, 8-7-7 setae in *B*-whorls, and 2-2-1 additional setae involved. All other genera of the tribe feature a complete set of distal setae; in addition, at least some of them, for instance *Detriturus jubilarius*, has *M*-seta (see fig. 389 in Pomorski 1998). These differences are evidently a good reason to complete the descriptions of tibiotarsal chaetotaxy in such oligochaetotic forms of Onychiurinae.

*Subdivision of sterna.* Among Thalassaphorurini there is a genus, *Spinonychiurus*, characterized by such a unique feature as a secondary division of *Abd*.3 sternum. Some traces of such division can also be seen in all well preserved specimens of *Sensillonychiurus* (Fig. 6), as well as in some other small-sized species of various group of Onychiurinae. Nevertheless, the anterior subsegment in *Sensillonychiurus* is narrow and, contrary to *Spinonychiurus*, lacks setae.

*Furcal remnant position.* In complete agreement with the main diagnostic character of Thalassaphorurini, the furcal remnant in all studied *Sensillonychiurus* is in the form of a finely granulated area in the mid-section of *Abd*.4, with four small setae arranged in two posterior rows. Individual variations in number and position of these setae are not frequent, but have been noted. The number of setal rows on manubrial area is also more or less stable: usually two rows (*mm* and *mp* according to Weiner (1996) with 4 setae in each can be distinguished (Fig. 33) although some variations especially in their position are also seen. Additionally 1+1 setae (*ma*?) usually present at a level with finely granulated area. The most significant is the anterior position of the latter area at contact with the border between *Abd*.3-4 sterna (Figs 6, 33). According to the available, mainly illustrative data (Fjellberg 1998, fig. 88A, Fig. 97F; Kaprus' and Stebaeva 2006, Fig. 7; Kaprus' and Tsalan 2009, Fig. 1.1), personal communication of R. Pomorski and our observations on the *volinensis*-group of *Allonychiurus*, all other genera of Thalassaphorurini are characterized by posterior position of furcal remnant in some distance from the border between *Abd*.3 and 4, and all of them (*flavescens*-group of *Allonychiurus* is the only exception) possess only one row of manubrial setae behind dental setae (Weiner 1996). These two characters, i.e. position of furcal remnant and number of manubrial rows of setae, clearly correlate. Thus, all studied species of the *volinensis*-group of *Allonychiurus* are characterized by the presence of the same number of 4+4 manubrial setae (Figs 43–44) as in *Sensillonychiurus* but due to posterior position of furcal remnant only one row of manubrial setae set posterior to dental setae. Species of the *flavescens*-group of *Allonychiurus* appear to be also characterized by posterior position of finely granulated area (see fig. 14 in Sun et al. 2010), but possess more manubrial setae arranged in several rows; sometimes a few additional setae are present (Weiner 1996; Sun et al. 2011). This difference was used as a main diagnostic feature in separation of *Allonychiurus* from *Tantulonychiurus* and *Thibaudichiurus* by Sun et al. (2011). However, it can also be considered as a result of polychaetosis clearly seen on fig. 1B in Sun et al. (2011). More investigation including a study of juveniles is probably needed to evaluate the significance of these differences.

*Anal spines*. A full spectrum from complete absence to strong spines set on low papillae is found among the studied species, but an intermediary situation is most frequent. The same is characteristic of *Spinonychiurus* and *Micronychiurus*, but not of *Detriturus* and *Agraphorura* (complete absence of spines), *Thalassaphorura* (*AS* absent as an exception) and *Allonychiurus* (spines always present).

Based on this review of the morphological features, the following can be concluded:

Regardless of one’s opinion on the status of the genus *Sensillonychiurus*, all studied species represent a rather homogeneous group of closely related forms, characterized by many common morphological features and seemingly congruent distributions mainly covering the northern parts of eastern Asia with insulated records from North America and Eastern Europe.

The genus *Sensillonychiurus* shares many characters with representatives of other genera of Thalassaphorurini, but a combination of characters seems to be unique for the tribe. The only features, which set the genus apart from all other Thalassaphorurini, appear to be not the number of guard setae in *AO* but dorsal chaetotaxy and anterior position of furcal remnant at a contact with border between *Abd*.3 and 4 although the data concerning other genera is still rather limited for a final decision.

Briefly, the genus can be defined as Thalassaphorurini featuring compound vesicles in the *PAO*, a partial reduction of guard setae in the *AO* and on the tibiotarsi, the absence of *d*0 on the head, anterior position of furca remnant and a clearly demarcated dorsal border between *Abd*.5 and 6.

### Sensillonychiurus

Pomorski & Sveenkova, 2006

http://species-id.net/wiki/Sensillonychiurus

#### Type-species.

*Sensillonychiurus minusculus* Pomorski & Sveenkova, 2006: 191, by original designation.

#### Diagnosis.

Small-sized Thalassaphorurini with low number of compound vesicles in PAO; labrum with 7 setae, labium of *AC* or *ABC*-type; *AO* with 4–5 papillae and 3–4 guard setae, smooth sensory clubs; distinct antennal, tergal and sternal sensilla, without *d*0 on head, *Abd*.4 with *m*0 and *p*0, *Abd*.5 with *p*0, *Abd*.6 dorsally with 1+1 prespinal microsetae and 1 medial macroseta; distal whorl of setae on *Ti*.1-3 with 7 or 9 setae, both *M* seta on all legs and *B*6 on *Ti*.3 absent; *pso* on *Th*.1 usually present, no tendency to dorsal *pso* multiplication, low number of sternal *pso*; *psx* usually absent; sternum of *Abd*.3 not clearly divided, furcal remnant situated at contact with border between *Abd*.3-4 sterna with two regular rows of manubrial setae set posteriorly to 4 dental setae; *AS* present or absent.

## Description of species

### Sensillonychiurus mirus sp. n.

urn:lsid:zoobank.org:act:E9A79C2A-7B38-405D-8C45-7B8E67C0C22C

http://species-id.net/wiki/Sensillonychiurus_mirus

Figs 1–7

#### Material.

 Holotype ♀, Russia, NW of European part, Kola Peninsula, Dalnie Zelentsy [69°07'N, 36°03'E ], coastal sandy steep with sparse vegetation (flotation), 19.vii.2009, leg. A. Babenko (MSPU).

Paratypes 5 ♀, same data as holotype (MSPU).

#### Description.

 Colour white. Size 0.56–0.60 mm. Body slender and elongated. Antennae about as long as head, antennal area not clearly demarcated. *Ant*.4 with two distinct thickened sensilla, subapical organite and basal microsensillum present (Fig. 1). *Ant*.3 organ consisting of 5 papillae, 2 sensory rods, 2 smooth and usually slightly bilobed sensory clubs (Fig. 2), 4 guard setae, and a lateral microsensillum (Fig. 1). *Ant*.1 and 2 with 7–8 and 12–13 setae, respectively. *PAO* with 7–8 composed vesicles (Fig. 3). Labrum with 7 setae and 4 prelabral ones. Apical part of labium with thick terminal setae on papillae *A*, *B* and *C* (*ABC*-type), 11 guard setae, *a*1 clearly longer and thicker than other spiniformguard setae, i.e. *b*1-2 and *d*2 (Fig. 4), and 5 proximal setae. Basal fields of labium (mentum and submentum) with 4 and 5 setae, hypostomal complex reduced to one long seta and a minute projection. Maxillary palp simple, with 2 sublobal setae.

Pseudocellar formula (*pso*) as follows, dorsal: 2(3)2/133/33343 (rarely some *pso* duplicated), ventral: 1/000/0000, parapseudocelli (*psx*) invisible. Each upper subcoxa with one *pso*. Localization of *pso* as in Fig. 5. Granulation fine and uniform, without areas of enlarged granules. Dorsal chaetotaxy almost symmetrical, setae smooth and clearly differentiated only on abdominal tip, in more anterior parts of body setae differing in shape but not in size: some of them straight, thick and blunt, others curved and pointed, sensilla distinct: 2/022/222221 (dorsal) and 2/000/00011 (ventral) (Figs 5–6), occasionally some additional mesosetae can be thickened and look like other sensilla, thickened sensillum present on coxae *Lg.*3 (Fig. 7). *Th*.1 with 6+6 setae as a rule. Lateral microsensilla present only on *Th*.2. Unpaired dorsal seta *d*0 on head absent, *Abd*.4 with *m*0 and *p*0, *Abd*.5 with *p*0, *Abd*.6 dorsally with one axial macroseta and 1+1 prespinal microsetae (Fig. 5). Thoracic sterna without setae along *linea ventralis*, ventral chaetotaxy of abdomen as in Fig. 6. *Abd*.3 sternum unclearly divided, anterior subsegment without setae. Furca reduced to a small area of fine granulation situated at contact with border between *Abd*.3-4 sterna, with 2+2 small posterior setae arranged in 2 rows, manubrial area with 4+4 setae set in two rows (Fig. 6). Ventral tube with 6+6 distal setae, proximal ones at corpus base absent. Upper subcoxae usually with 3-3-4, tibiotarsi with 17-17-16, setae: distal whorl with 9 setae (7 *A* and two *T*-setae), row *B* with 7-7-6 setae, setae *M* absent but *Y* present. Unguis simple, with neither inner nor lateral tooth, unguiculus with an indistinct basal lamella, shorter than unguis (Fig. 7). Anal spine short but rather strong, set on unclear papillae.

#### Affinities.

*Sensillonychiurus mirus* sp. n. clearly differs from the all previously described species of the genus first of all in having not three but four guard setae in *AO*. Nevertheless it is not a unique character for the group. The same structure of *AO* (5 papillae and 4 guards) as in *Sensillonychiurus mirus* sp. n. is known in two other species of the genus, *Sensillonychiurus vitimicus* sp. n. and *Sensillonychiurus amuricus* sp. n. (see descriptions below). All these species which are characterized by only a weak reduction of *AO* with a highest possible number of papillae and 4 guard setae have many other characteristics in common (see Table 1.). Nonetheless, *Sensillonychiurus mirus* sp. n. can easily be distinguished from *Sensillonychiurus vitimicus* sp. n. by the complete absence of setae on thoracic sterna, from *Sensillonychiurus amuricus* sp. n. in the different type of labium (*ABC* in *Sensillonychiurus mirus* sp. n. versus *AC* in *Sensillonychiurus amuricus* sp. n.), and in four prelabral setae (*Sensillonychiurus amuricus* sp. n. possesses only two prelabral setae which are more common in the genus).

**Table 1.** Main diagnostic characters of the known species of *Sensillonychiurus*

	Dorsal pso	Dorsal sensilla	AO papillae/guards	Position of ms on Ant.4	Number of prelabral setae	Type of labium	ms on Th.3	Number of setae on Th.1	Ventral setae on thorax	Number of distal tibiotarsal setae	pso/psx on Abd.4	Unguiculus / unguis ratio	Anal spines
*Sensillonychiurus eisi*	32/133/33343	1/011/22211	4/3	low	?	AC	?	5+5	?	9	?	0.3	–
*Sensillonychiurus minusculus*	32/133/33343	1/011/222111	4/3	low	2	AC	+	5+5	–	7	–	0.5	–
*Sensillonychiurus virginis*	32/022/33343	1/011/222111	4/3	low	?	AC	–	5+5	–	9	–	0.33	–
*Sensillonychiurus geminus*	32/133/33343	1/011/222111	5/3	low	?	AC	+	5+5	–	9	pso	0.75	+
*Sensillonychiurus mirus* sp.n.	2(3)2/133/33343	2/022/222221	5/4	upper	4	ABC	–	6+6	–	9	–	~0.5	+
*Sensillonychiurus taimyrensis* sp.n.	32/133/33343	1/011/221111	4/3	upper	2	AC	–	6+6	–	7	–	~0.6	+
*Sensillonychiurus vegae* sp.n.	32/133/33343	1/011/221111	4/3	low	2	AC	–	6+6	–	7	–/psx	~0.6	+
*Sensillonychiurus vitimicus* sp.n.	32/133/33343	1/011/221111	5/4	upper	4	ABC	–	6+6	+	9	–	~0.6	+
*Sensillonychiurus amuricus* sp.n.	32/133/33343	1/011/221111	5/4	upper	2	AC	–	6+6	–	9	–	~0.7	+
*Sensillonychiurus* sp.	32/133/33343	1/011/2222?11	4/3	low	2	?	–	6+6	–	9	–	?	+

#### Etymology.

 Initially, the name *mirus* (odd, strange, unusual in Latin) reflects both an isolated position of the new species within the genus and the gap between its type-locality and the distributions of the other known species of the genus which are pure Asiatic or American. The level of morphological uncommonness has lowered after the performed survey of all available material, but the geographical isolation still exists.

#### Distribution.

 Known only from the type locality.

### Sensillonychiurus taimyrensis sp. n.

urn:lsid:zoobank.org:act:AC031C4C-13EA-45F9-9DD5-575BD8287653

http://species-id.net/wiki/Sensillonychiurus_taimyrensis

Figs 8 10–14, 18, 21

#### Material.

 Holotype ♀, Russia, Taimyr Peninsula, northern coast of Taimyr Lake, Postoyannaya River [74°38'N, 101°55'E ], low river terrace, mosses, *Dryas* sp., *Astragalus* spp., 02.viii.1993, leg. A. Babenko (MSPU).

Paratypes 5 ♀ and 4 ♂, same data as holotype; 2 ♀ and 1 ♂, Taimyr Peninsula, northwestern coast of Lake Pyasino [70°04'N, 87°39'E ], herbaceous meadow on south-facing slope, sand, 03.viii.2001; 16 ♀, 10 ♂ and 6 juv., Taimyr Peninsula, middle reaches of Pyasina River, Ust-Tareya [73°15'N, 90°35'E ], herbaceous meadow on south-facing slope, 22.vii.2010, leg. A. Babenko (MSPU).

Other material. 1 ♀, Russia, Siberia, northwestern Buryatia, Ust’-Barguzin [53°25'N, 109°01'E ], Lake Baikal shore, sandy beach (ca 5 m from water edge, flotation), 21.viii.2008, leg. M. Potapov; 1 ♀, Russia, Siberia, Buryatia, Vitim Plateau, vicinity of Eravna (Sosnovo-Ozerskoe) [52°27'N, 111°09'E ], dry birch forest, 21.viii.2009, leg. A. Chimitova.

#### Description.

**Figures 10–23.**
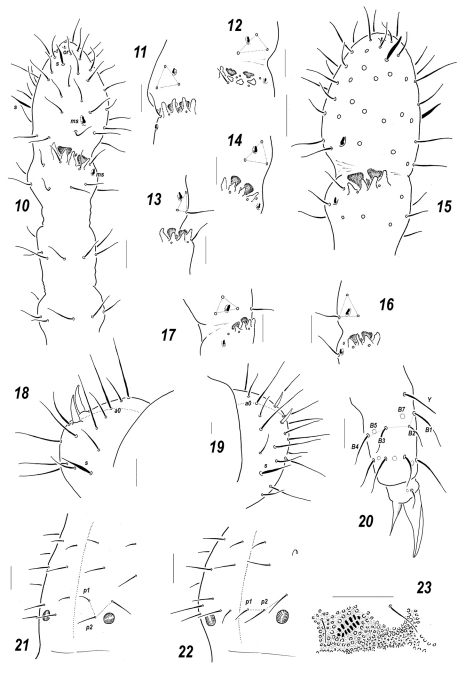
*Sensillonychiurus taimyrensis* sp. n. **(10–14, 18, 21)** and *Sensillonychiurus vegae* sp. n. **(15–17, 19–20, 22–23)** **10** antenna **11–17** position of *ms* on *Ant*.4, different views **18–19** *Abd*.6 **20** tibiotarsus of *Lg*.3; **21–22** axial chaetotaxy of *Abd*.3 **23** ventral *psx* on posterolateral part of *Abd*.4 (specimen from Vitim Plateau). Scale: 0.01 mm.

 Colour white. Size 0.56–0.62 mm. Body slender and elongated. Antennae about as long as head, antennal area not clearly demarcated. *Ant*.4 with a subapical organite, two distinct thickened sensilla, and a subbasal microsensillum set well above proximal row of setae (Figs 10–14). *Ant*.3 organ consisting of 4 papillae, 2 sensory rods, 2 smooth sensory clubs, 3 guard setae, and a lateral microsensillum (Fig. 10). *Ant*.1 and 2 usually with 8 and 13 setae, respectively. *PAO* with 7(8) composed vesicles. Labrum with 7 setae and 2 prelabral ones (2/3-4). Apical part of labium of *AC*-type, with (5)6 proximal setae and usually with a complete set of guard setae (11), although asymmetrical absence of one of *e*-guard setae also visible, *a*1-guard long. Basal fields of labium (mentum and submentum) with 4 and 5 setae. Hypostomal complex with one long and one shorter projection. Maxillary palp simple, with 2 sublobal setae.

Pseudocellar formula (*pso*) as follows, dorsal: 32/133/33343, ventral: 1/000/0000, parapseudocelli (*psx*) invisible. Each upper subcoxa with one *pso*. Localization of *pso* as in Fig. 8. Granulation rather fine and uniform, without areas of clearly enlarged granules. Dorsal chaetotaxy almost symmetrical, setae smooth and clearly differentiated, especially on last abdominal terga, in anterior parts of body meso and microsetae similar in size but differing in shape: mesosetae straight and blunt, microsetae curved and pointed, sensilla more or less distinct on terga and less evident on sterna: 1/011/221-2111 (dorsal) (Fig. 8) and 2/000/0000-1 (ventral), sensillum on coxae of *Lg.*3 present but not distinct. *Th*.1 with 6+6 setae. Lateral microsensilla present only on *Th*.2. Unpaired dorsal seta *d*0 on head absent, *Abd*.4 with *m*0 and *p*0, *Abd*.5 with *p*0, *Abd*.6 with one axial macroseta (Figs 8, 18). Axial microsetae *p*1 set anteriorly to mesosetae *p*2 on *Abd*.1-3 (Fig. 21). Thoracic sterna without setae along *linea ventralis*. *Abd*.3 sternum unclearly divided, anterior subsegment without setae. Furca reduced to a small area of fine granulation situated at contact with border between *Abd*.3-4 sterna, with 2+2 small posterior setae arranged in two rows, manubrial area with 4+4 setae set in two rows. Ventral tube with 6+6 distal setae, proximal ones at corpus base absent. Upper subcoxae usually with 3-4-4, tibiotarsi with 15-15-14, setae: distal rows with 7 setae (all *T*-setae absent), row *B* with 7-7-6 setae, setae *M* absent but *Y* present. Unguis simple, with neither inner nor lateral tooth, unguiculus with an indistinct basal lamella, clearly shorter than unguis (about 0.6–0.65 *U*3).Anal spines short (0.7–0.75 *U*3) but rather thick (thickness/length 0.23–0.28), set on low papillae.

#### Affinities.

 Apart from *Sensillonychiurus taimyrensis* sp. n., only two known species of the genus, i.e. *Sensillonychiurus minusculus* and *Sensillonychiurus vegae* sp. n., completely lack all *T*-setae on tibiotarsi (distal whorl with 7 setae). *Sensillonychiurus minusculus* clearly differs in having lateral *ms* on *Th*.3 and *Abd*.6 without *AS*. Two other species, *Sensillonychiurus vegae* sp. n. and *Sensillonychiurus taimyrensis* sp. n. are very similar, sharing many common characteristics (see Table 1). Nonetheless *Sensillonychiurus taimyrensis* sp. n. can be easily distinguished due to stronger *AS* set on low papillae (cf. Figs 18 and 19), more distal position of *ms* on *Ant*.4 (cf. Figs 10–14 and 15–17) and clear differences in the mutual position of microsetae *p*1 and mesosetae *p*2 on *Abd*.3 (cf. Figs 21 and 22).

#### Etymology.

 The new species was named after its *terra typica*.

#### Distribution.

 Despite a few records the new species is probably widespread in eastern Siberia being found in such remote regions as Taimyr’s tundras and mountainous Buryatia. Previously the species was erroneously listed for Taimyr as *Tantulonychiurus volinensis* (Szeptycki, 1964) by Babenko (2003) and Babenko and Fjellberg (2006).

### Sensillonychiurus vegae sp. n.

urn:lsid:zoobank.org:act:0086C2ED-D20C-45F3-A220-4F4D9383558B

http://species-id.net/wiki/Sensillonychiurus_vegae

Figs 9 15–17, 19–20, 22–23

#### Material.

 Holotype ♂, Russia, eastern Siberia, Yakutia (Sakha Republic), mouth of Yana River, Shirokostan Peninsula, vicinity of Lake Ledyanoe [72°25'N, 141°00'E ], *Dryas* association on steep slope, 04.viii.1994, leg. A. Babenko (MSPU).

Paratypes 6 ♀, 1 ♂, and 1 juv., Russia, eastern Siberia, Yakutia (Sakha Republic), left bank of Kolyma River [69°32'N, 160°44'E ], grass (*Elymus sibiricus*) association on a polar fox hill, 19.viii.1994, leg. A. Babenko (MSPU).

Other material: 1♀ and 2♂, Russia, Siberia, northwestern Buryatia, Ust’-Barguzin [53°25'N, 109°01'E ], shore of Lake Baikal, pine forest on sandy dunes (flotation), 21.viii 2008, leg. M. Potapov; 2♀, 6♂ and 6 juv., Russia, Siberia, Buryatia, Vitim Plateau, vicinity of Eravna (Sosnovo-Ozerskoe) [52°27'N, 111°09'E ], pine forest with *Rhododendron dauricum*, 08.ix.2008, leg. A. Chimitova; 2 ♂, same region, but birch forest, 25.viii.2009, leg. A. Chimitova (MSPU).

#### Description.

 Colour white. Size 0.40–0.52 mm, holotype 0.47 mm long. Body slender and elongated. Antennae about as long as head, antennal area not clearly demarcated. Sensillar armature of *Ant*.4 as usual: two distinct thickened sensilla, a subapical organite and a basal microsensillum set almost in line with proximal row of setae (Figs 15–17). *Ant*.3 organ consisting of 4 papillae, 2 sensory rods, 2 smooth sensory clubs, 3 guard setae, and a lateral microsensillum (Fig. 15). *Ant*.1 and 2 usually with 8 and 13(14) setae, respectively. *PAO* with 6–7(8) composed vesicles. Labrum with 7 setae and 2 prelabral ones (2/3–4), four setae of apical row thicker. Apical part of labium with thick terminal setae on papillae *A* and *C* (*AC* – type), (5)6 proximal setae and a complete set (11) of guard setae: 7 long [*b*3-4, *d*3-4, *e*1-3] and 4 spiniform [*a*1, *b*1-2 and *d*2] ones set on papillae, *a*1 clearly longer than others. Basal fields (mentum and submentum) with 4 and 5 setae. Maxillary palp simple, with two sublobal setae.

Pseudocellar formula (*pso*) as follows, dorsal: 32/133/33343, ventral: 1/000/0000, *Abd*.4 sterna with or without 1+1 parapseudocelli laterally (see Variability). Each upper subcoxa with one *pso*. Granulation fine and uniform, slightly enlarged granules rarely present around medial *pso* on abdominal tip and on head. Dorsal chaetotaxy almost symmetrical (Fig. 9), setae smooth and clearly differentiated, especially on last abdominal terga, in anterior parts of body meso and microsetae only slightly differing in size but different in shape: mesosetae straight and blunt, microsetae curved and pointed. Tergal sensilla (1/011/221111 in number) distinct, sternal ones (2/000/0000-1) hardly distinguished, sensillum on coxae of *Lg.*3 evident. *Th*.1 usually with 6+6 setae. Lateral microsensilla present only on *Th*.2. Unpaired dorsal seta *d*0 on head absent, *Abd*.4 with *m*0 and *p*0, *Abd*.5 with *p*0, *Abd*.6 with one axial macroseta (Figs 9, 18). Axial microsetae *p*1 lying almost in line with mesosetae *p*2 on *Abd*.3 (Fig. 22) and sometimes also on *Abd*.2. Thoracic sterna without setae along *linea ventralis*. *Abd*.3 sternum unclearly divided, anterior subsegment narrow and without setae. Furca reduced to a small area of fine granulation situated at contact with border between *Abd*.3-4 sterna, with 2+2 small posterior setae arranged in two rows, manubrial area usually with 4+4 setae set in two rows. Ventral tube with 6+6 distal setae, proximal ones at corpus base absent. Upper subcoxae usually with 3-4-4, tibiotarsi with 15-15-14, setae: distal rows with 7 setae (all *T*-setae absent), row *B* with 7-7-6 setae, setae *M* absent but *Y* present (Fig. 20). Unguis simple, with neither inner nor lateral tooth, unguiculus with an indistinct basal lamella, about 0.6 times as long as inner edge of *U*3.Anal spine rather long (0.6–0.7 *U*3) but thin (thickness/length 0.13–0.23) (Fig. 19), set without papillae.

#### Variability.

The types of *Sensillonychiurus vegae* sp.n. completely lack *psx* as well as all so far studied species of the genus. Nonetheless, at least some of the specimens collected on Vitim Plateau possess 1+1 ventral parapseudocelli on *Abd*.4 (Fig. 23) being otherwise identical to the types. This population may represent a separate species, but its reliable distinction is hardly possible. Anyway, more material from different points of the distributional range is needed to evaluate the constancy and significance of this character.

#### Affinities.

 Virtually all of the main morphological characteristics of *Sensillonychiurus vegae* sp. n. (structure of *AO* and *PAO*, labrum and labium, dorsal and ventral chaetotaxy, number and distribution of *pso*, presence of *ms* only on *Th*.2, number of setae on subcoxae, tibiotarsi and *VT*) are identical to those of sympatric *Sensillonychiurus taimyrensis* sp. n. Concerning the differences of *Sensillonychiurus vegae* sp. n. from *Sensillonychiurus taimyrensis* sp. n. see description of the latter.

#### Etymology.

 The new species was initially collected during the joint Swedish-Russian expedition arranged in 1994 in order to commemorate A.E. Nordenskiöld’s first trip on “Vega” board along the Northern Sea Route (1878–1879). That is why it is named after Nordenskiöld’s famous steamship “Vega”.

#### Distribution.

 Known from several remote areas of eastern Siberia. Previously, it was erroneously listed for Yakutia as *Tantulonychiurus volinensis* (Szeptycki, 1964) by Babenko and Fjellberg (2006).

### Sensillonychiurus amuricus sp. n.

urn:lsid:zoobank.org:act:E99FF1BA-7739-4019-A89B-7F2E998A520F

http://species-id.net/wiki/Sensillonychiurus_amuricus

Figs 24–31

#### Material.

 Holotype ♀, Russia, Asiatic part, Khabarovsk suburbs, right bank of Amur river [48°33'N, 135°01'E ], upper part of sandy beach (flotation), 26 iv 2010, M. Potapov leg (MSPU).

Paratypes 3 ♀, 4 ♂ and 1 juv., same data as holotype (MSPU).

#### Description.

 Colour white. Size of mature specimens 0.62–0.72 mm. Body slender and elongated. Antennae about as long as head, antennal area not clearly demarcated. *Ant*.4 with a subapical organite, two distinct thickened sensilla, and a subbasal microsensillum set well above proximal row of setae (Fig. 25). *Ant*.3 organ consisting of 5 papillae, 2 sensory rods, 2 smooth sensory clubs (Figs 26–27), 4 guard setae, and a lateral microsensillum (Fig. 25). *Ant*.1 and 2 usually with 8 and 13(14) setae, respectively. *PAO* with 6–7 composed vesicles (Fig. 28). Labrum with 7 setae and 2 prelabral ones (2/3–4). Apical part of labium with thick terminal setae on papillae *A* and *C* (*AC* – type), 7 long guard setae [*b*3-4, *d*3-4, *e*1-3] and 4 spiniform ones [*a*1, *b*1-2 and *d*2] set on low papillae, *a*1 clearly longer and thicker than *b*1. Proximal part of labium as usual, with 6 setae, basal fields (mentum and submentum) with 4 and 5 setae. Maxillary palp simple, with 2 sublobal setae.

**Figures 24–31.**
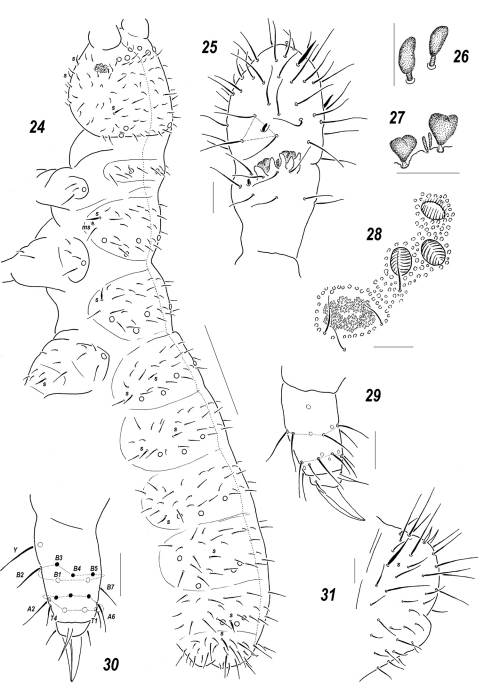
*Sensillonychiurus amuricus* sp. n. **24** dorsal chaetotaxy **25** *Ant*.3–4 **26–27** sensorial elements of *Ant*.3 organ, different view **28** *PAO* and adjacent *pso* **29–30** tibiotarsus of *Lg*.3, different views **31** *Abd*.6. Scales: 24 – 0.1 mm, 25–31 – 0.01 mm.

Pseudocellar formula (*pso*) as follows, dorsal: 32/133/33343, ventral: 1/000/0000, parapseudocelli (*psx*) invisible. Each upper subcoxa with one *pso*. Localization of *pso* as in Fig. 24. Granulation rather fine and uniform, without areas of clearly enlarged granules. Dorsal chaetotaxy almost symmetrical, setae smooth and clearly differentiated, especially on last abdominal terga, differences between macro- and microsetae in anterior parts of body not so pronounced but visible: macrosetae more straight and blunt, microsetae curved and pointed. Dorsal sensilla distinct, flame-like, 1/011/221111 in number (Fig. 24), ventral ones (2/000/0001) slightly thickened and sometimes hard to detect, sensillum on coxae of *Lg.*3 distinct. *Th*.1 with 6+6 setae. Lateral microsensilla present only on *Th*.2. Unpaired dorsal seta *d*0 on head absent, *Abd*.4 with *m*0 and *p*0, *Abd*.5 with *p*0, *Abd*.6 with one axial macroseta (Fig. 24). Thoracic sterna without setae along *linea ventralis*. *Abd*.3 sternum unclearly divided, anterior subsegment without setae. Furca reduced to a small area of fine granulation situated at contact with border between *Abd*.3-4 sterna, with 2+2 small posterior setae arranged in two rows, manubrial area with 4+4 setae set in two rows. Ventral tube with 6+6 distal setae, proximal ones at corpus base absent. Upper subcoxae usually with 3-4-4, tibiotarsi with 17-17-16, setae: distal rows with 9 setae (2 *T*-setae absent), row *B* with 7-7-6 setae, setae *M* absent but *Y* present (Figs 29–30). Unguis simple, with neither inner nor lateral tooth, unguiculus with an indistinct basal lamella, shorter than unguis (ca 0.7 *U*3).Anal spines short (0.7–0.75 *U*3) and thin, set without papillae (Fig. 31).

#### Affinities.

 The same structure of *AO* (five papillae and four guard setae) as in *Sensillonychiurus amuricus* sp. n. is only known in two species of the genus, *Sensillonychiurus mirus* sp. n. and *Sensillonychiurus vitimicus* sp. n. All these species which are characterized by only a weak reduction of *AO* with a full number of papillae and 4 guard setae also show the highest number of setae (9) in the distal tibiotarsal whorl. Both can easily be distinguished from *Sensillonychiurus amuricus* sp. n. in having a different type of the labium (*ABC* versus *AC* in *Sensillonychiurus amuricus* sp. n.) and four prelabral setae (*Sensillonychiurus amuricus* sp. n. possesses only two prelabral setae, which occurs more commonly in the genus). Apart from this, *Sensillonychiurus amuricus* sp. n. is the largest congener.

Two other species of the genus, *Sensillonychiurus virginis* and *Sensillonychiurus geminus*, are characterized by the most complete set of tibiotarsal setae (17-17-16) but against the background of a pronounced reduction of *AO.*

#### Etymology.

 The new species was named after its *terra typica*.

#### Distribution.

 Known only from the type locality.

### Sensillonychiurus vitimicus sp. n.

urn:lsid:zoobank.org:act:1EB550A9-8192-4BEF-8826-93D1ABB96418

http://species-id.net/wiki/Sensillonychiurus_vitimicus

Figs 32 36

#### Material.

 Holotype ♂, Russia, Siberia, Buryatia, Vitim Plateau, vicinity of Telemba [52°44'N, 113°16'E ], larch forest with *Betula fruticosa*, 23.viii.2009, leg. A. Chimitova (MSPU).

Paratypes 7 ♀ and 3 ♂, same data as holotype; 1♀ same region but… larch forest with rich herbaceous cover, 04.x.2009, leg. A. Chimitova (MSPU).

#### Description.

 Colour white. Size 0.58–0.68 mm (females), 0.50–0.58 (males). Body slender and elongated. Antennae about as long as head, antennal area not clearly demarcated. *Ant*.4 with 2 distinct thickened sensilla, a subapical organite and a basal microsensillum present, the latter set well above proximal row of setae (Fig. 34). *Ant*.3 organ consisting of 5 papillae, 2 sensory rods, 2 smooth sensory clubs, 4 guard setae, and a lateral microsensillum (Fig. 34). *Ant*.1 and 2 with 8 and (12)13 setae, respectively. *PAO* with 7–8 composed vesicles. Labrum with 7 setae and 4 prelabral ones. Labium of *AC-*type, but terminal setae on papillae *C* slightly thinner, guard setae as usual for genus: 7(6) long (*b*3-4, *d*3-4, *e*1-3) and 4 spiniform (*a*1, *b*1-2 and *d*2) ones, *a*1 clearly longer and thicker than others. Proximal part of labium with (5)6 setae, mentum and submentum with 4 and 5 setae, respectively. Maxillary palp simple, with 2 sublobal setae.

**Figures 32–33.**
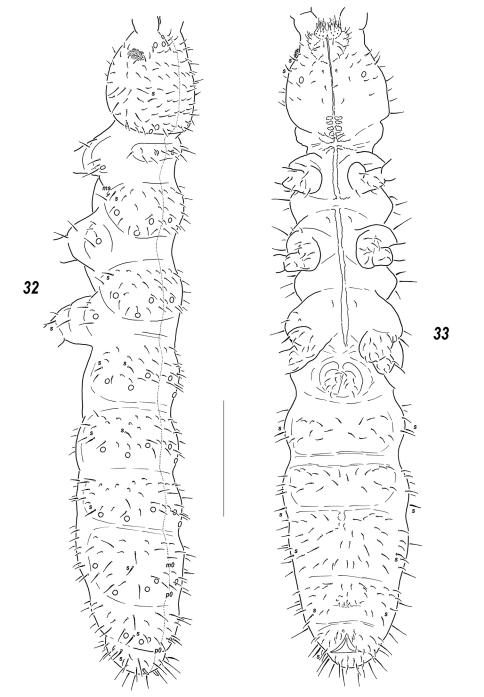
*Sensillonychiurus vitimicus* sp. n. **32** dorsal chaetotaxy **33** ventral chaetotaxy. Scale: 0.1 mm.

Pseudocellar formula (*pso*) as follows, dorsal: 32/133/33343, ventral: 1/000/0000, parapseudocelli (*psx*) invisible. Each upper subcoxa with one *pso*. Localization of *pso* as in Fig. 32. Granulation fine and uniform, slightly enlarged granules often present around *pso* on last abdominal terga. Dorsal chaetotaxy almost symmetrical, setae smooth and clearly differentiated especially on abdominal tip, in more anterior parts of body macro and microsetae mainly differing in shape, sensilla distinct on terga and less evident on sterna: 1/022/221111 (dorsal) and 2/000/00011 (ventral) (Figs 32–33), thickened sensillum present also on coxae of *Lg.*3. *Th*.1 with 6+6 setae. Lateral microsensilla present only on *Th*.2. Unpaired dorsal seta *d*0 on head absent, *Abd*.4 with *m*0 and *p*0, *Abd*.5 with *p*0, *Abd*.6 dorsally with one axial macroseta and 1+1 prespinal microsetae (Fig. 32). Thoracic sterna with 0-1-1 setae on each side of *linea ventralis*, ventral chaetotaxy of abdomen as in Fig. 33. *Abd*.3 sternum unclearly divided, anterior subsegment without setae. Furca reduced to a small area of fine granulation situated at contact with border between *Abd*.3-4, with 2+2 small posterior setae arranged in two rows, manubrial area with 4+4 setae set in two rows (Fig. 33). Ventral tube with 6+6(7) distal setae, proximal ones at corpus base absent. Upper subcoxae usually with 3-(3)4-4, tibiotarsi with 17-17-16 setae: distal rows with 9 setae (7 *A* and two *T*-setae), row *B* with 7-7-6 setae, setae *M* absent but *Y* present (Fig. 36). Unguis simple, with neither inner nor lateral tooth, unguiculus with indistinct basal lamella, clearly shorter than unguis (Fig. 36).Anal spine rather strong (about as long as 0.6–0.7 *U*3), set on unclear papillae (Fig. 35).

**Figures 34–39.**
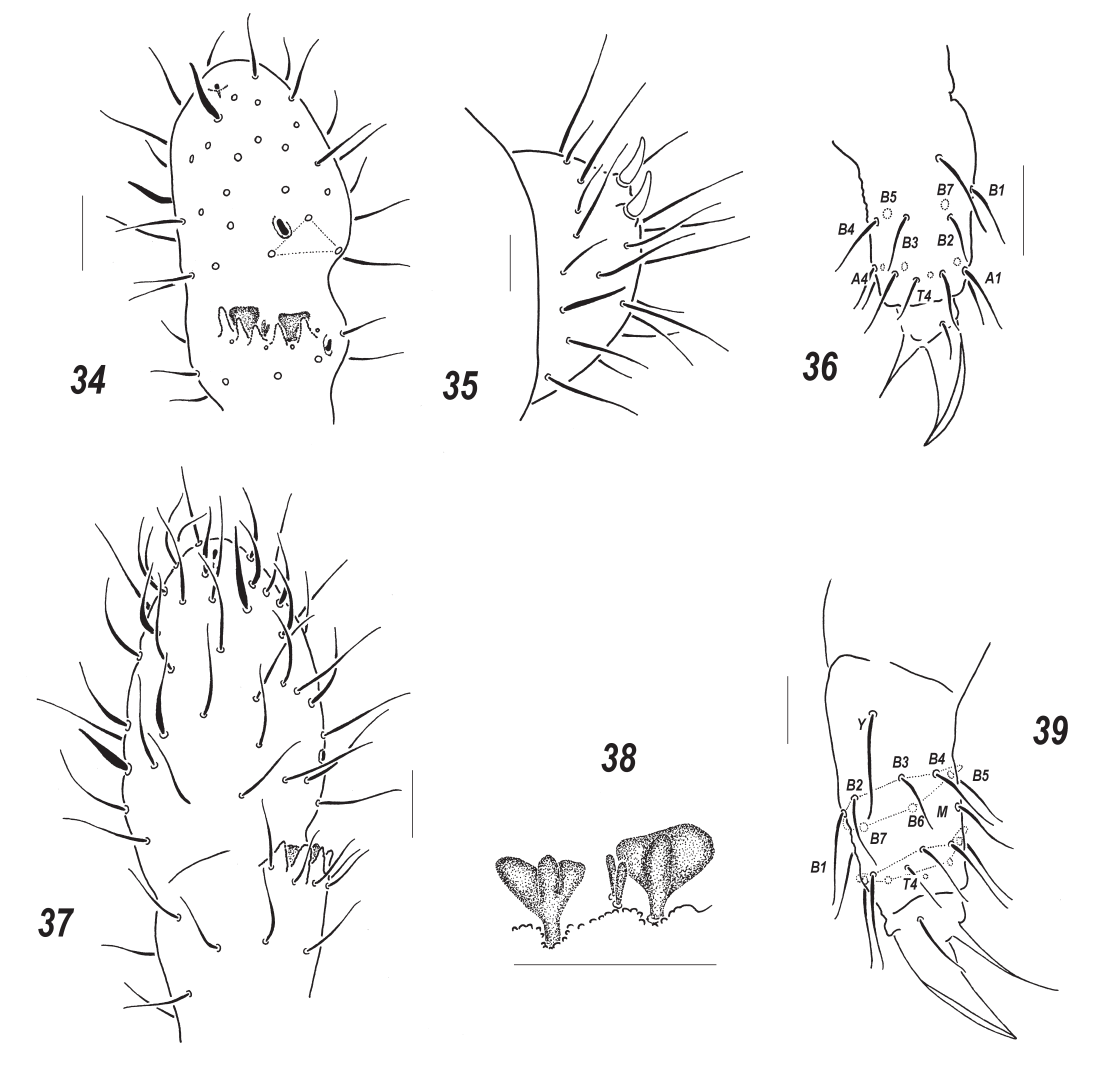
*Sensillonychiurus vitimicus* sp. n. **(34–36)** and *Allonychiurus elikonius* sp. n. **(37–39)** **34, 37** *Ant*.3-4 **35** *Abd*.6 **36, 39** tibiotarsus of *Lg*. 3, different views **38** sensorial elements of *Ant*.3 organ. Scale: 0.01 mm.

#### Affinities.

 Due to the presence of four guard setae in *AO*, *Sensillonychiurus vitimicus* sp. n. is the most similar to *Sensillonychiurus mirus* sp. n. and *Sensillonychiurus amuricus* sp. n. All these three species have many other characteristics in common (see Table 1), but *Sensillonychiurus vitimicus* sp. n. can easily be distinguished by the presence of setae on thoracic sterna (a presumed apomorphic condition within Onychiuridae according to Fjellberg (1998).

#### Etymology.

 The new species was named after its *terra typica*.

#### Distribution.

 Known from several biotopes in vicinity of the type locality.

One more species of the genus *Sensillonychiurus* was found on Kamchatka (vicinity of Petropavlovsk, sandy sea beach with weed debris, leg. L. Lobkova). It differs from *Sensillonychiurus virginis* in having setiform anal spines, from *Sensillonychiurus geminus* by the absence of lateral *ms* on *Th*.3. The lack of material (only a single female is available) did not allow us to describe it, but it is listed in the key and in Table 1 as *Sensillonychiurus* sp.

##### Key to the known species of Sensillonychiurus Pomorski & Sveenkova, 2006

**Table:** 

1	*AS* not differentiated	2
–	*AS* present	4
2	Tibiotarsi with 7 distal setae	*Sensillonychiurus minusculus* Pomorski & Sveenkova, 2006
–	Tibiotarsi with 9 distal setae	3
3	Dorsal *pso* as 32/022/33343	*Sensillonychiurus virginis* Pomorski & Sveenkova, 2006
–	Dorsal *pso* as 32/133/33343	*Sensillonychiurus eisi* (Rusek, 1976), comb. n.
4	Tibiotarsi with 9 distal setae	5
–	Tibiotarsi with 7 distal setae	9
5	Both *Th*.2-3 with lateral *ms*, ventral *pso* on *Abd*.4 present [1/000/0101 as a whole]	*Sensillonychiurus geminus* Pomorski & Sveenkova, 2006
–	Only *Th*.2 with lateral *ms*, *Abd*.4 without ventral *pso* [1/000/0000 as a whole]	6
6	*AO* with 5 papillae and 4 guard setae (Figs 1, 25)	7
–	*AO* with 4 papillae and 3 guard setae (as in Fig. 10)	*Sensillonychiurus* sp.
7	Thorax with ventral setae	*Sensillonychiurus vitimicus* sp. n.
–	Thorax without ventral setae	8
8	Labium of the *ABC* type (Fig. 4), 4 prelabral setae present	*Sensillonychiurus mirus* sp. n.
–	Labium of the *AC* type, only two prelabral setae present	*Sensillonychiurus amuricus* sp. n.
9	*AS* strong, set on low papillae (Fig. 18), *ms* on *Ant*.4 clearly above proximal setae (Figs 10–14), microsetae *p*1 set anteriorly to mesosetae *p*2 on all terga from *Abd*.1 to *Abd*.3 (Fig. 21)	*Sensillonychiurus taimyrensis* sp. n.
–	*AS* as thick short setae (Fig. 19), *ms* on *Ant*.4 almost in line with proximal setae (Figs 15–17), microsetae *p*1 set in line with *p*2 on *Abd*.3 (Fig. 22)	*Sensillonychiurus vegae* sp. n.

### Genus Allonychiurus

Yosii, 1995

http://species-id.net/wiki/Allonychiurus

Pseudonychiurus Lin, 1980 (mistakenly created for the moulting specimen) (synonym)
Tantulonychiurus Pomorski, 1996, syn. n.
Thibaudichiurus Weiner, 1996 (synonym)

#### Type-species.

*Onychiurus flavescens* Kinoshita, 1916: 458, by original designation.

#### Diagnosis.

Small- or medium-sized Thalassaphorurini with compound vesicles in PAO; labrum with 7 or 9 setae, labium of *AC* or *ABC*-type; *AO* with 4–5 papillae and 5 guard setae, smooth or granulated sensory clubs; antennal and tergal sensilla usually distinct, *d*0 on head present, *Abd*.4 and 5 usually with some axial microsetae, *Abd*.6 dorsally with 2+2 prespinal microsetae and 1–2 medial macrosetae; distal whorl on *Ti*.1-3 with 7, 9 or 11 setae, *B-*whorlusually complete on all tibiotarsi*, M* seta present; no tendency to dorsal *pso* multiplication, head and abdominal sterna with ventral *pso*, dorsal *pso* on *Th*.1 usually present; *psx* not numerous or absent; sternum of *Abd*.3 not subdivided, furcal remnant situated at some distance from border between *Abd*.3-4 sterna, with one or several rows of manubrial setae posterior to dental setae; *MVO* present or absent; *AS* present.

#### Remarks.

 As it was already stressed in Introduction the genus is accepted here in a wider scope than it was proposed by Sun et al. (2011) to include species described below. In this scope the genera *Tantulonychiurus* Pomorski, 1996 and *Thibaudichiurus* Weiner, 1996recognized as valid by Sun et al. (2011) are placed here in synonymy of *Allonychiurus*. In our opinion, the generic value of main differentiated character of these genera, i.e. the number of setal rows on manubrial area, appears to be size and age dependent and needs further attention to be proved. The genus in the accepted scope is rather heterogeneous but completely analogous to *Thalassaphorura* which mainly differs in having simple vesicles in *PAO*. Here we only deal with the representatives of so called *volinensis*-group of the genus characterized by small size (less than 1.0 mm), smooth sensorial clubs and usually four papillae in *AO*. According to the generic classification proposed by Sun et al. (2011), the species described below should probably be assigned to the genus *Tantulonychiurus* since all of them are characterized by only one row of manubrial setae posterior to dental microsetae and by the position of *MVO* on *Abd*.4 sternum if present. In this case, the degrees of reduction of the tibiotarsal setae found in the new species completely fill up the gap between *Tantulonychiurus* and *Thibaudichiurus* (7–9 versus 9 setae, respectively) and make their distinction rather problematic, taking also into account that not all of these species possess a *MVO*. That is why we are inclined to leave a decision concerning the status of all these genera pending a complete revision of the complex.

### Allonychiurus elikonius sp. n.

urn:lsid:zoobank.org:act:6843EC79-00D9-4039-96E1-D5088E2ACA99

http://species-id.net/wiki/Allonychiurus_elikonius

Figs 37 44

#### Material.

 Holotype ♀, Russia, Yakutia (Sakha Republic), Suntar-Khayata Mt Range, upper reaches of Kyubyume River [63°13'N, 139°32'E ], 1,300 m alt., sandbank in Elikon River bed (flotation), 06.vii.2002, leg. O. Makarova (MSPU).

Paratypes: 22 females on slides and more than 300 specimens in alcohol, same data as holotype; 11 females, same region, 1,480 m alt., plant community with predominance of *Dryas* sp. on slope, 07.vii.2002; 7 females, same region, 1,430 m alt., herbaceous meadow on south-facing slope, 07.vii.2002; 14 females on slides and more than 800 specimens in alcohol, same region, greenhouse of “Vostochnaya” Meteorological Station, 1,287 m alt., 24.vii.2002, leg. O. Makarova (MSPU).

#### Description.

 Colour white. Size 0.72–0.84 mm. Body slender and elongated. Antennae about as long as head, antennal area not clearly demarcated. *Ant*.4 rather long and narrow, with several curved and slightly thickened sensilla, 2 of which (dorso-subapical and inner-subbasal) straighter and especially distinct, a subapical organite small, usually spherical, a basal microsensillum present (Fig. 37). *Ant*.3 organ consisting of 4 (or rarely 4+5) low papillae, 2 sensory rods, 2 smooth sensory clubs with ribs (Fig. 38), 5 guard setae, and a lateral microsensillum (Fig. 37). *Ant*.1 and 2 as a rule with 9 and 12–13 setae. *PAO* with 10–12 composed vesicles set at some distance from each other (Fig. 42). Labrum with 7 setae and 4 prelabral ones. Apical part of labium with thick terminal setae on papillae *A* and *C* (*AC*-type), 6 long (*b*3-4, *d*3-4, *e*1, 3; *e*2 absent) and 4 spiniform (*a*1, *b*1-2 and *d*2), guard setae, *a*1shorter than others (Fig. 41). Proximal field of labium usually with 6 setae, basal fields (mentum and submentum) with 4 and 6 setae. Maxillary palp simple, with 2 sublobal setae.

**Figures 40–44.**
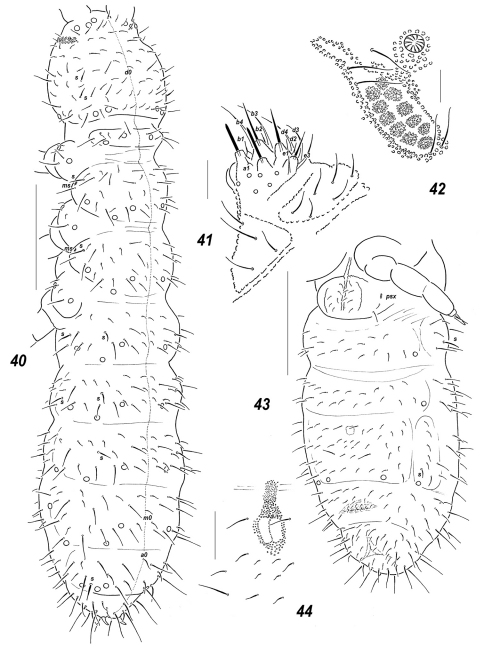
*Allonychiurus elikonius* sp. n. **40** dorsal chaetotaxy **41** labium **42** *PAO* and adjacent *pso* **43** ventral chaetotaxy **44** furcal remnant. Scales: 40, 43 – 0.1 mm, 41-42, 44 – 0.01 mm.

Pseudocellar formula (*pso*) as follows, dorsal: 32/233/33343, ventral: 11/000/0112, additionally one parapseudocellus (*psx*) present on each side of *VT* anteriorly to basal setae (Fig. 43). Each upper subcoxa with two *pso*. Localization of *pso* as in Figs 40, 43. Granulation fine and uniform, without areas of enlarged granules. Dorsal chaetotaxy almost symmetrical, setae smooth and clearly differentiated, especially on abdominal tip, sensilla not always distinct, sometimes hard to detect, particularly so on sterna and medially on *Abd*.1-3: 2/011/222010 (dorsal) and 2/000/00010 (ventral) (Fig. 40), a thickened sensillum on coxae of *Lg.*3 present. *Th*.1 with 5-6(7) setae on each side. Terga of *Th*.2-3 and *Abd*.1-3 with 3+3, *Abd*.4 with 2+2 and *Abd*.5 with 1+1, axial microsetae. Lateral microsensilla present on both *Th*.2-3. Unpaired dorsal setae: *d*0 on head, microseta *m*0 on *Abd*.4, microseta *a*0 on *Abd*.5, and 2 macrosetae *a*0 and *m*0 on *Abd*.6, supplemented by 2+2 prespinal microsetae (Fig. 40).

Sterna of *Th*. 2-3 with 1+1 setae along *linea ventralis*, ventral chaetotaxy of abdomen as in Fig. 43. *Abd*.3 sternum unclearly divided, anterior subsegment without setae. Furca reduced to a small area of fine granulation situated at some distance from border between *Abd*.3-4, with 2+2 small posterior setae arranged in 2 rows (Fig. 44), manubrial area with 4+4 setae arrange in 2 rows but only one of them set posteriorly to small dental setae (Fig. 43). Ventral tube with (5)6+6 distal setae and 2 proximal ones at corpus base. Upper subcoxae with (3)4-4-4, tibiotarsi with 18-18-18, setae: distal whorl with 9 setae (7 *A* and 2 *T*-setae), 7 setae in row *B* on each leg, setae *M* and *Y* present (Fig. 39). Unguis simple, with neither inner nor lateral tooth, unguiculus narrow with a long apical filament, latter usually reaching slightly beyond unguis (Fig. 39).Anal spine thick and slightly curved, set on unclear papillae.

#### Affinities.

**Figures 45–49.**
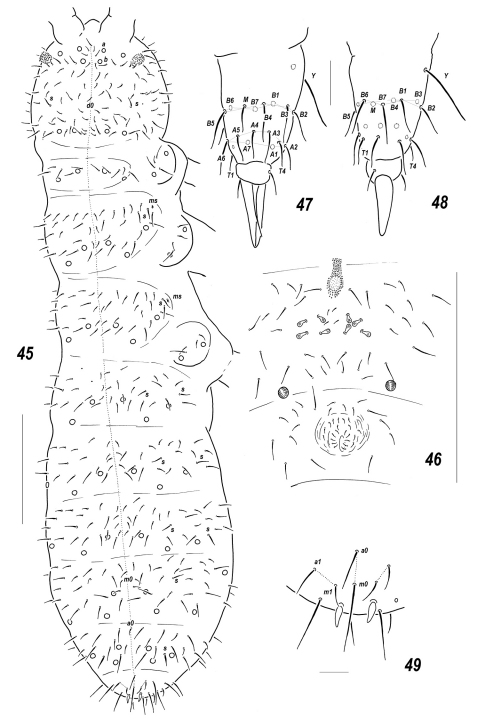
*Allonychiurus subvolinensis* sp. n. **45** dorsal chaetotaxy **46** *MVO* on *Abd*.4 **47–48** tibiotarsus of *Lg*.3, different views **49** *Abd*.6, dorsal chaetotaxy. Scales: 45–46 – 0.1 mm, 47–49 – 0.01 mm.

 The main morphological features of *Agraphorura elikonius* sp. n. are similar to those of *Agraphorura volinensis*, *Agraphorura subvolinensis* sp. n. and *Agraphorura asiaticus* (Babenko, 2007), comb. n. (see Table 2). Thus, all four species are characterized by virtually identical dorsal chaetotaxy and similar numbers of *pso* on all terga, sterna and subcoxae. The presence of a complete set of *B*-setae and *M*-seta on all tibiotarsi is also shared by them. *Agraphorura elikonius* sp. n. has a different type of the labium (*AC* in *Agraphorura elikonius* sp. n. versus *ABC* in three other species) and differs from *Agraphorura volinensis* and *Agraphorura subvolinensis* in the mutual position of antennal *pso* (cf. Figs 40 and 45). There are also some variations of the number of distal setae on the tibiotarsi in these four species (7 setae in *Agraphorura volinensis* and *Agraphorura asiaticus*, 9 in *Agraphorura elikonius* and *Agraphorura subvolinensis*). *Agraphorura asiaticus* is the only species in the group showing five papillae in *AO* (found in *elikonius* only in exceptional cases), and only *Agraphorura subvolinensis* is characterized by the presence of setae on all thoracic sterna (absent from *Th*.1 in all other species).

**Table 2.** Main diagnostic characters of the known species of the *volinensis*-group of *Allonychiurus*

	Dorsal pso	Ventral pso	pso on upper subcoxae	AO papillae/ guards	Ventral setae on thorax	Dorsal sensilla	Type of labium	Number of prelabral setae	Number of distal setae on tibiotarsi	Unguiculus / unguis ratio	MVO position	Number of setae on VT
*Allonychiurus volinensis*	32/233/33343	11/000/1112‡	2-2-2	4/5	0-1-1	1/011/222121	ABC	4	7	0.9-1.1	Abd.4	1/6/2
*Allonychiurus foliatus*	32/233/33323†	01/000/0000 [?]	1-2-2	4/5	?	?	?	?	?	0.75	VT + genital plate	?/6/?
*Allonychiurus mariangeae*	32/233/33343	11/000/1112	2-2-2	4/5	1-1-1	2/011/111111	?	?	9	0.75	genital plate	0/6/2
*Allonychiurus donjiensis*	22/222/22222 [?]	11/200/0011 [?]	1-1-1	4/5	?	?	?	?	?	0.75	?	?/6/?
*Allonychiurus jindoensis*	32/233/33333	10/000/0102	1-1-1	4/5	?	?	?	?	?	0.75	?	?/6/?
*Allonychiurus asiaticus*	32/233/33343	11/000/0112	2-2-2	5/5	1-1-1	1/011/222221	ABC	4	7	0.7-0.8	absent	1/6/2
*Allonychiurus elikonius* sp. n.	32/233/33343	11/000/0112	2-2-2	4(5)/5	0-1-1	2/011/222010	AC	4	9	0.9-1.1	males unknown	0/6/2
*Allonychiurus subvolinensis* sp. n.	32/233/33343	11/000/1112	2-2-2	4/5	1-1-1	1/011/222111	ABC	4	9	~0.9	Abd.4	1/6/2
*Allonychiurus unisetosus* sp.n.	32/233/33343	11/000/0111	2-2-2	4/5	0-1-1	1/011/222121	ABC	2	9	0.9-1.1	Abd.4 [?]	1/6/2

† According to the original description, the species is characterized by 33/233/33323 dorsal *pso* and complete absence of ventral *pso*; most lateral *pso* on posterior side of a head are considered here as being ventral.‡ Slightly different formula of ventral *pso*, i.e. 11/000/0112, is given by Fjellberg (1998)

It is more difficult to distinguish *Allonychiurus elikonius* sp. n. from three Korean and one Chinese species of the group, namely *Allonychiurus mariangeae* (Thibaud & Lee, 1994), *Allonychiurus donjiensis* (Lee & Kim, 1994), *Allonychiurus jindoensis* (Lee & Kim, 1994), and *Allonychiurus foliatus* (Rusek, 1967), because their descriptions are incomplete and probably not fully correct in certain details. The most complete description is that of *Allonychiurus mariangeae*. It is rather similar to *Allonychiurus elikonius* sp. n. in having an almost identical chaetotaxy, the same number of dorsal *pso* and tibiotarsal setae (see Table 2). The only difference of the sternal *pso* formula is the presence of true pseudocellus on *Abd*.1 in *Allonychiurus mariangeae* instead of an elongated parapseudocellus without clear cuticular ring in *Allonychiurus elikonius* sp. n. However, these organs are homologous and sometimes difficult to distinguish. The most characteristic feature of *Allonychiurus mariangeae* is the presence of *MVO* in mature males. Unfortunately, *Allonychiurus elikonius* sp. n. in the region under study is only represented by parthenogenetic populations: among more than 100 specimens checked, all were females. Formally, these species differ in size (0.75–0.83 mm in *Allonychiurus elikonius* sp. n. versus 0.5–0.65 mm in *Allonychiurus mariangeae*), in the absence of ventral setae on *Th*.1 in *elikonius*, in the different number of setae on *Ant*.1 (9 in *Allonychiurus elikonius* versus 8 in *Allonychiurus mariangeae*), by unguiculus length (equal to or slightly longer than unguis in *Allonychiurus elikonius* versus 0.75 of *U*3 in *Allonychiurus mariangeae*), and by the absence of *a*0 on *Abd*.5 in *Allonychiurus mariangeae*, but all these characters are probably variable.

Three remaining species of the *volinensis*-group were described as having a lesser number of dorsal and ventral *pso* (see Table 2). Yet this probably needs verification. In any case, clear differences in the ecological preferences of compared species confirm the specificity of *Allonychiurus elikonius* sp. n. The monsoon subtropical climate of southern Korea (the habitats of *Allonychiurus mariangeae*, *Allonychiurus donjiensis*, and *Allonychiurus jindoensis* are sand beaches) and central China (vicinity of Shanghai, the only known locality of *Allonychiurus foliatus*) has nothing to do with the extremely continental conditions of mountainous Yakutia (about 160 km from Oymyakon, one of the coldest places on Earth), where *Allonychiurus elikonius* sp. n. was found. Nevertheless, the probability that some of these nominate species can probe to be conspecific with *Allonychiurus elikonius* sp. n. cannot be completely ruled out until their adequate redescriptions.

#### Etymology.

 The new species was named after its type-locality, Elikon River.

#### Distribution.

 Still known only from the region of the type-locality, where it inhabits a number of different communities up to 1,500 m alt.

### Allonychiurus subvolinensis sp. n.

urn:lsid:zoobank.org:act:2400049E-3FC5-4642-AD7D-05683CA7F275

http://species-id.net/wiki/Allonychiurus_subvolinensis

Figs 45–49

#### Material.

 Holotype ♂, Russia, Tuva Republic, northern macroslope of Eastern Tannu-Ola Mt Range, 5 km S of Lake Chagytai [51°00'N, 94°43'E ], larch forest belt, 1,300 m alt., under larch (*Larix sibirica*), 16.vi.2003, leg. S.K. Stebaeva (MSPU).

Paratypes ♂, same region and locality, ca 1,400 m, 17.vi.2003; ♀, same region, meadow steppe, ca 1,200 m alt., under *Dracocephalum ruyschiana*, 17.vi.2003; 6♀ and 3♂, Russia, Tuva Republic, southern macroslope of Eastern Tannu-Ola Mt Range, 20 km N of Khol’-Oozhu [50°44'N, 94°23'E ], 1,600 m alt., meadow steppe, under *Spiraea* sp., 16.vii.1993; 7 specimens, Russia, Tuva Republic, foothills of southern macroslope of Eastern Tannu-Ola Mt Range, basin of Aryskannyg-Khem River, 15 km E of Khol’-Oozhu [50°41'N, 94°35'E ], ca 1,100-1,250 m alt., dry steppe, under *Nanophyton grubovii*, 17.vii.1993; 12♀ and 10♂, Russia, Tuva Republic, Sangelen Plateau, 25–30 km NE of Erzin [50°15'N, 95°09'E ], ca 1,000 m alt., upper terrace of Erzin River, steppe with *Caragana spinosa*, 03viii.1995, all leg. S.K. Stebaeva (MSPU).

#### Description.

 Colour white. Size 0.55–0.62 mm. Body slender and elongated, slightly wider in region of *Abd*.4. Antennae about as long as head, antennal area not clearly demarcated. *Ant*.4 rather short and wide, 2 usual sensilla not especially thickened but distinct, a subapical organite and a basal microsensillum present. *Ant*.3 organ consisting of 4 low papillae, 2 sensory rods, 2 smooth sensory clubs without clear ribs, 5 guard setae, and a lateral microsensillum. *Ant*.1 and 2 with 8 and (12)13 setae, respectively. *PAO* wide (length/width ratio ca 1.5), with about 7–10 composed vesicles set close together. Labrum as a rule with 7 setae and 4 prelabral ones, but holotype with an abnormal number of setae set asymmetrically. Apical part of labium with thick terminal setae on papillae *A*, *B* and *C* (*ABC* – type), seta *A* clearly thicker, 6 long (*e*2 absent) and four spiniform (*a*1, *b*1-2 and *d*2), guard setae, *a*1shorter than others. Proximal field of labium with 5 setae, basal fields (mentum and submentum) with 4 and 5 setae. Maxillary palp simple, with 2 sublobal setae.

Pseudocellar formula (*pso*) as follows, dorsal: 32/233/33343 (Fig. 45), ventral: 11/000/1112. Each upper subcoxa with two *pso*. Granulation fine and uniform, without areas of enlarged granules. Dorsal chaetotaxy more or less symmetrical, setae smooth and rather thick, clearly differentiated only on abdominal tip, sensilla: 1/011/222111 (dorsal) and 2/000/00010 (ventral), but distinguishable mainly because of their stable positions, only lateral ones on *Th*.2-*Abd*.1 and posterior one on *Abd*.5 always distinct (Fig. 45), as well as a sensillum on coxae of *Lg.*3. *Th*.1 with 5+5(6) setae. Terga of *Th*.2-*Abd*.1 with 3, *Abd*.2-3 with 3(4), *Abd*.4 with 2-3 and *Abd*. 5 with 1, pairs of axial microsetae, additionally each tergum with 1+1 posterior axial mesosetae set slightly out of line with microsetae. Some unpaired dorsal setae also present: *d*0 on head, microseta *m*0 on *Abd*.4, microseta *a*0 on *Abd*.5, and two macrosetae *a*0 and *m*0 on *Abd*.6, supplemented by 2+2 prespinal microsetae (Fig. 45, 49). Lateral microsensilla present on both *Th*.2-3.

Each sternum of *Th*. 1-3 with 1+1 setae along *linea ventralis*. Secondary division of *Abd*.3 sternum unclear because of bad preservation. Furca reduced to a small area of fine granulation situated at some distance from border between *Abd*.3-4 sterna with 2+2 small posterior setae arranged in 2 rows (Fig. 46), manubrial area with 4+4 setae arrange in 2 rows but only one of them set posteriorly to dental setae (Fig. 46). Ventral tube usually with 1+1 frontal, 6+6(5–7) distal and 2 proximal setae at corpus base. Upper subcoxae usually with 4-4-4, tibiotarsi with 18-18-18. setae: distal row on each leg with 9 setae (7 *A* and 2 *T*-setae), 7 setae in row *B*, setae *M* and *Y* present (Figs 47–48). Unguis simple, with neither inner nor lateral tooth, unguiculus narrow, almost as long as unguis (ca 0.9 *U*3).Anal spines thick and slightly curved, set without clear papillae. Reproductive males with *MVO* identical to that in *Allonychiurus volinensis* with 4+4 modified club-like setae in mid-ventral section of *Abd*.4 behind furcal remnant (Fig. 46), in not reproductive males these setae spiniform.

#### Affinities.

*Allonychiurus subvolinensis* sp. n. is very similar to the European *Allonychiurus volinensis* (Szeptycki, 1964), comb. n. in many features. Both have a somewhat isolated position within the *volinensis*-group of *Allonychiurus* due to the wide *PAO*, the presence of *MVO* on *Abd*.4 and the different positions of *pso* at the antennal base, with *b*-pseudocelli set closer to the mid-line than *a*-pseudocelli. They can easily be distinguished from each other due to the different number of tibiotarsal setae (9 setae in distal whorl in *Allonychiurus subvolinensis* sp. n. versus 7 setae in *Allonychiurus volinensis*) and by the presence of ventral setae on all thoracic sterna in *Allonychiurus subvolinensis* sp. n. (*Allonychiurus volinensis* lacks setae on *Th*.1). The third very similar species of the same group, *Allonychiurus unisetosus* sp. n., is described below. For differences with *Allonychiurus volinensis* and *Allonychiurus subvolinensis* sp. n. see the description of *Allonychiurus unisetosus* sp. n.

#### Etymology.

 The name reflects the general similarity to *Allonychiurus volinensis*.

#### Distribution.

 The new species was previously listed for Tuva as *Onychiurus* s.str. by Stebaeva (2003). It appears to be rather common in the region in various plant communities, from mountain forests to arid steppes.

### Allonychiurus unisetosus sp. n.

urn:lsid:zoobank.org:act:613014D9-6782-4406-B0C6-CEC3DEEDB568

http://species-id.net/wiki/Allonychiurus_unisetosus

Figs 50–54

#### Material.

 Holotype ♂, Russia, Tuva Republic, northern macroslope of Eastern Tannu-Ola Mt Range, vicinity Shuurmak [50°38'N, 95°18'E ], spruce-larch (*Picea obovata*, *Larix sibirica*) forest, on larch stump under *Cladonia chlorophaea*, 1,450 m alt., 12.viii.1997, leg. N.V. Sedel'nikova (MSPU).

Paratypes 8♀ and ♂, same sample as holotype; 1♀, same region, stony outcrops in mountain steppe, under *Xanthoparmelia somloёnsis* and *Parmelia saxatilis*, 1,450 m alt., 12.viii.1997, leg. N.V. Sedel'nikova (MSPU).

#### Description.

 Colour white. Size 0.55–0.65 mm. Body elongated, wider in region of *Abd*.4. Antennae about as long as head, antennal area not clearly demarcated. *Ant*.4 rather short and wide, 2 usual sensilla not especially thickened but distinct, a subapical organite and a basal microsensillum present. *Ant*.3 organ consisting of 4 low papillae, 2 sensory rods, 2 smooth sensory clubs without clear ribs, 5 guard setae, and a lateral microsensillum (Fig. 52). *Ant*.1 and 2 usually with 8 and 13 setae, respectively. *PAO* wide (length/width ratio ca 1.5), with (7)8–10 composed vesicles set close together. Labrum with 7 setae and 2 prelabral ones. Apical part of labium with thick terminal setae on papillae *A*, *B* and *C* (*ABC* – type), seta *A* clearly thicker, 6 long (*e*2 absent) and four spiniform (*a*1, *b*1-2 and *d*2), guard setae, *a*1shorter than others. Proximal field of labium with 5 setae, basal fields (mentum and submentum) with 4 and 5 setae. Maxillary palp simple, with 2 sublobal setae.

**Figures 50–54.**
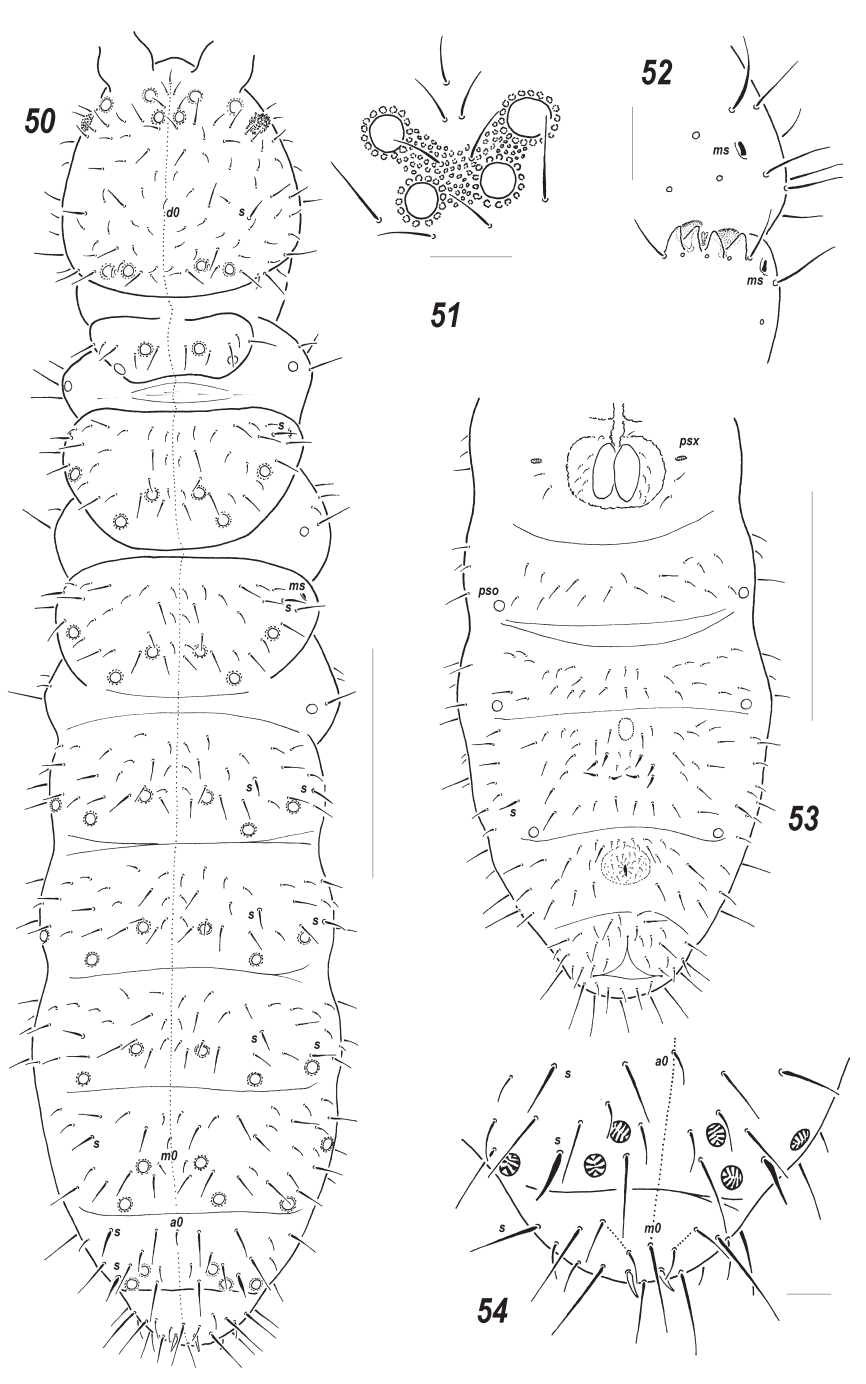
*Allonychiurus unisetosus* sp. n. **50** dorsal chaetotaxy **51** position of anteromedial *pso* on head **52** *AO* **53** ventral chaetotaxy of abdomen **54** *Abd*.6, dorsal chaetotaxy. Scales: 50, 53 – 0.1 mm, 51–52, 54 – 0.01 mm.

Pseudocellar formula (*pso*) as follows, dorsal: 32/233/33343 (Fig. 50), ventral: 11/000/0111 (one specimen with 1+2 ventral *pso* on *Abd*.4 also visible), sternum of *Abd*.1 with 1+1 *psx* on each side of *VT* (Fig. 53). Upper subcoxae with two *pso* and (2)3-(3)4-(3)4 setae, respectively. Generally granulation rather fine, but areas of clearly enlarged granules usually present around some *pso* and in mid and lateral parts of thorax. Dorsal chaetotaxy almost symmetrical, setae smooth and rather thick, clearly differentiated into macro and microsetae, sensilla poorly distinguishable, 1/011/222121 (dorsal) and 2/000/00010 (ventral), only lateral ones on *Th*.2-*Abd*.1 and posterior one on *Abd*.5 always distinct (Figs 50, 54). Sensillum on coxae of *Lg.*3 present. *Th*.1 with (4)5+5 setae. Terga of *Th*.2-*Abd*.3 with 3, *Abd*.4 with 2 and *Abd*. 5 with 1, pairs of axial microsetae, additionally each tergum with 1+1 posterior axial mesosetae set slightly out of line with microsetae. Unpaired dorsal setae: *d*0 on head, microseta *m*0 on *Abd*.4, microseta *a*0 on *Abd*.5, and only one macrosetae (*m*0) on *Abd*.6, supplemented by 2+2 prespinal microsetae (Figs 50, 54). Lateral microsensilla present on both *Th*.2-3.

Sterna of *Th*. 1-3 with 0-1-1 setae on each side of *linea ventralis*. Furca reduced to a small area of fine granulation situated at some distance from border between *Abd*.3-4 sterna with 2+2 small posterior setae arranged in 2 rows, manubrial area with 4+4 setae arrange in 2 rows but only one row set posteriorly to small dental setae (Fig. 53). Ventral tube usually with 1+1 frontal, 6+6 distal and 2(3) proximal setae at corpus base. Tibiotarsi with 18-18-18 setae: distal row on each leg with 9 setae (7 *A* and 2 *T*-setae), 7 setae in row *B*, setae *M* and *Y* present. Unguis simple, without teeth, unguiculus narrow, gradually tapering, with fine filament reaching tip of unguis. Anal spines curved and rather thin, set without papillae. *MVO* in reproductive males probably identical to that in *Allonychiurus volinensis* but in both available mature males only thickened setae present in mid-ventral section of *Abd*.4 (Fig. 53).

#### Affinities.

*Allonychiurus unisetosus* sp. n., *Allonychiurus volinensis* and *Allonychiurus subvolinensis* sp. n. constitutes a rather homogeneous subgroup among the known species of the *volinensis*-group of *Allonychiurus*. All of them are characterized by identical position of antennal *pso* with *b*-pseudocellus set close to midline and out of antennal area (cf. Figs 50–51 and 40). Such a position is unique for the group. *Allonychiurus unisetosus* sp. n. shares equal number of tibiotarsal setae (9) with *Allonychiurus subvolinensis* sp. n. and identical ventral chaetotaxy of thorax (0-1-1) with *Allonychiurus volinensis* (see Table 2) but differs from both species in having only two prelabral setae, one ventral *pso* on *Abd*.4 as a rule, only one axial macroseta on dorsal side of *Abd*.6 (cf. Figs 54 and 49), and clearly thinner *AS*.

#### Etymology.

 The name reflects the presence of only one axial macroseta on *Abd*.6 in the new species separating it from similar congeners.

#### Distribution.

 Known from several nearby localities of mountain Tuva, previously listed for the same region as *Onychiurus* s.str. sp. by Stebaeva et al. (2001).

### Allonychiurus asiaticus

(Babenko, 2007) comb. n.

http://species-id.net/wiki/Allonychiurus_asiaticus

Tantulonychiurus asiaticus Babenko 2007 (synonym)

#### Material.

 15 specimens, Russia, Siberia, Krasnoyarsk Territory, Achinsk Region, 7 km from Nazarovo [57°02'N, 90°39'E ], ca 400 m alt., meadows of various types, 1987–88; 9 specimens, Russia, West Siberia, 25 km S of Novosibirsk, Academgorodok [54°49'N, 83°08'E ], wet grass-herbaceous meadow, 02.X.1994, all leg. S. Stebaeva.

The above new material collected from an area lying far south (more than 1,000 km) of the terra typica of the species differs from the original description in having more clearly differentiated tergal sensilla, but otherwise being very similar. These specimens may even represent a separate species, but material from intermediate areas is needed to evaluate the significance of these differences.

## Supplementary Material

XML Treatment for Sensillonychiurus

XML Treatment for Sensillonychiurus mirus

XML Treatment for Sensillonychiurus taimyrensis

XML Treatment for Sensillonychiurus vegae

XML Treatment for Sensillonychiurus amuricus

XML Treatment for Sensillonychiurus vitimicus

XML Treatment for Allonychiurus

XML Treatment for Allonychiurus elikonius

XML Treatment for Allonychiurus subvolinensis

XML Treatment for Allonychiurus unisetosus

XML Treatment for Allonychiurus asiaticus
